# Insights into Nonelectroactive C–C Bond Formation on Cu(100) during Electrochemical CO_2_ Reduction from Multiconfigurational Wavefunction Theory

**DOI:** 10.1021/acs.jpcc.5c07792

**Published:** 2026-02-27

**Authors:** John Mark P. Martirez, Emily A. Carter

**Affiliations:** † 17217 Princeton Plasma Physics Laboratory, Princeton, New Jersey 08543-0451, United States; ‡ Department of Mechanical and Aerospace Engineering and the Andlinger Center for Energy and the Environment, Princeton University, Princeton, New Jersey 08544-5263, United States

## Abstract

Carbon–carbon (C–C) bond formation is necessary for hydrocarbon (and oxygenate) synthesis beyond methane (and formate/formic acid) during electrochemical CO and CO_2_ reduction (ECOR and ECO_2_R). Cu has notable ability to form hydrocarbons compared to other pure metals. In particular, the (100) facet of face-centered cubic Cu forms ethylene competitively with H_2_ and methane during both ECOR and ECO_2_R. Past simulations based on density functional theory (DFT) with standard exchange-correlation functional approximations predict fast nonelectroactive C–C bond formation channels involving adsorbed (*) CO together with another *CO, formyl (*CHO), or hydroxy­methylidyne (*COH), forming OC*–*CO, OC*–CHO*, and OC*–*COH, respectively. Such simulations support the prevailing hypothesis that emergence of C_2_ products is kinetically determined at the early stages of the reduction chemistry. Here we show, via simulations with more accurate many-body, i.e., “correlated”, wavefunction theory (enabled by an embedding scheme), that the coupling of *CO with a *CO or a *COH (previously predicted at the same level of theory to kinetically dominate over *CHO as the one-electron reduction product of *CO) is highly activated (kinetically impeded), with free energy barriers >1 eV, in contradiction to previous DFT-based simulations. Intriguingly, we find that the coupling of two adjacent *COHs incurs only a small barrier (<0.3 eV) and is exoergic (< –1 eV); however, given the predicted low surface mobility of *COH, the emergence of HOC*–*COH is also improbable, at least at low *COH coverages. We therefore conclude that it is highly unlikely for *CO to participate in *nonelectroactive* C–C bond formation on pristine Cu(100), contrary to conventional wisdom, and that the energetically favorable *COH dimerization may occur only after substantial buildup of *COH on the surface.

## Graphical abstract

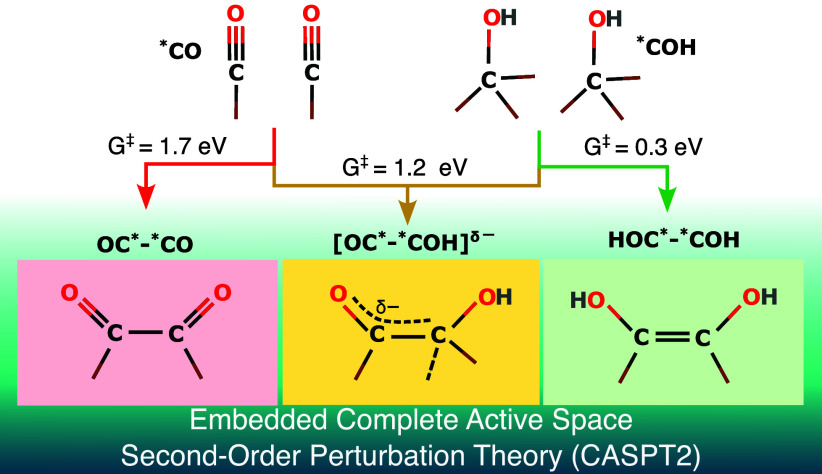

## Introduction

Interest in studying electrochemical CO and CO_2_ reduction (ECOR and ECO_2_R) presses on as society continues to seek ways to remove and then store or convert carbon dioxide that society emits as we consume fossil fuels for energy and generation of industrial chemicals.^1–3^ Electrochemical CO_2_ conversion’s appeal stems from the idea that, if it can be done efficiently, we can enable economically advantageous synthesis of fuels or chemical precursor products from CO_2_ while using electricity generated via carbon-free nuclear or renewable energy sources, thus contributing to the creation of a sustainable circular carbon economy.^4,5^ Such a lofty goal warrants extensive R&D to achieve commercialization and many fundamental questions remain that need to be addressed before this technology truly advance.^3–5^ One of the key concerns is the limited ability of metal cathodes to catalyze hydrocarbon formation. Among pure metals, only Cu is able to meaningfully produce such molecules, and even then, the Faradaic efficiency (selectivity) is low for anything other than methane (CH_4_) and ethylene (C_2_H_4_), with concomitant low formation rates.^2,6^ Despite Cu’s limited activity, there is value in understanding how Cu achieves such a feat that other metals cannot. This understanding is especially valuable as we chemically engineer new catalysts for ECOR and ECO_2_R that can produce or coproduce industrially relevant hydrocarbon molecules in large quantities.

Experiments have shown that CO_2_ conversion to CO on Cu is not rate-limiting.^7,8^ For many metals, CO is one of the major terminal products of ECO_2_R, e.g., Au, Ag, and Pd.^6^ On Cu, CO is deemed a critical intermediate that determines rate and selectivity of both ECOR and ECO_2_R. Notably, C–C bond formation in the early steps of ECO/CO_2_R on Cu is deemed crucial in eventually forming hydrocarbon molecules with two C atoms or more (C_2+_).^2^ The activity of Cu is facet dependent^9^ and the ability of the Cu(100) facet to preferentially form ethylene C_2_H_4_ over CH_4_, alongside H_2_ (the main product), is noteworthy (the opposite is true for Cu(111)).^10,11^ Adsorbed CO, *CO (* next to C means adsorbed via the C atom; we will use this nomenclature hereafter), is a critical species in ECO/CO_2_R. *CO has two possible one-electron–one-proton reduction products, namely, *COH (hydroxy­methylidyne) and *CHO (formyl). Many experimental (mostly without direct proof)^10,12–14^ and density functional theory (DFT)-based^14–22^ (mostly on pristine surfaces) studies proposed that coupling of *CO, *COH, *CHO, or their combinations, are possible precursors of C_2_H_4_.

Among the more direct attempts to identify a C_2_ intermediate on Cu(100) is the work of Perez-Gallent et al. that claimed to have identified the emergence of OC*—*COH during ECOR on Cu(100) using Fourier transform infrared (FTIR) spectroscopy.^23^ Their conclusion is based on the existence of 1191 and 1677 cm^–1^ band peaks in H_2_O (not observed on Cu(111)), which they assigned to the stretching modes of ^12^C—OH and ^12^C=O of monomeric *CO at a hollow site. The band peaks increase in intensity from +0.1 to −0.2 V vs the reversible hydrogen electrode (RHE), although no ethylene is expected to form within this range.^23^ After using D_2_O instead of H_2_O, they then observed a ^12^C=O stretching mode at 1584 cm^–1^, which they claimed to be not from monomeric *CO (the band is presumably masked in H_2_O because O—H bending modes occur within 1650–1450 cm^–1^). They then used DFT to justify IR band assignments, where they calculated the vibrational frequencies of *CO (hollow site), *COH, *CHO, *OC*—CO, lithiated OC*—*CO, OC*—*COH, OC*—*CHO, and HOC*—*COH. Neglecting the point that neither *CHO nor *COH could possibly form at the potential range they conducted their experiments, the authors claimed the peaks are from OC*—*COH where C atoms at bridge sites exhibited DFT-calculated C—OH and C=O stretch frequencies of 1235 and 1576 cm^–1^.^23^ We posit that the authors’ interpretations were limited by their assumption that *CO reduction to *COH (or *CHO) is rate-determining and their belief a priori that the DFT-based mechanisms are more or less correct, both of which affected their down selection and assignment process. In brief, because the authors already assumed that what they observed could only be from the dimerization of *CO, *COH, or *CHO, then their experiments would be interpreted as such. Kim et al. also claimed evidence of direct *CO coupling on highly defective sprayed powder Cu using time-resolved attenuated total-reflection surface-enhanced infrared absorption spectroscopy and proposed that coupling only occurs at grain boundaries.^24^ Beyond these two experiments, no other spectroscopic data are available, to our knowledge. Therefore, the identity of the relevant C_2_ intermediate, even on just pristine Cu(100), is far from being experimentally resolved.

Atomic-scale simulations offer an opportunity to “view” and understand these crucial coupling steps that are hard, if not impossible, to detect experimentally. Many quantum-mechanics-based studies use DFT with approximate exchange and correlation (XC) functionals to calculate reaction free energies and barriers of possible elementary steps. Notable examples of DFT-based simulations of C–C coupling during ECOR on Cu(100) includes work by Montoya et al.,^16^ where the authors showed with DFT and the PBE exchange-correlation (XC) functional (hereafter DFT-PBE) that inclusion of a nearly full monolayer of water molecules may markedly stabilize OC*–*CO at bridge sites. The presence of water reduces the energy barrier of coupling of two *COs at adjacent bridge sites from ∼1.0 eV, absent water and without a clear energy-minimum at the product state, to ∼0.4 eV and a product that is a local minimum with water present, although the reaction remains endothermic (∼0.3 eV). In the same paper, the authors also showed that explicit excess negative charge, generated by introducing species that self-ionize into a cation and electron in the Cu slab, can further reduce the barrier (to ∼0.3 eV) and even make the reaction exothermic (∼ –0.1 eV). They further showed that the nature of the compensating cation (H_3_O^+^, Li^+^, or Na^+^) does not meaningfully affect the energetics of coupling.

Luo et al.^17^ investigated the influence of applied potential on the ECO/CO_2_R elementary reactions using DFT-PBE in conjunction with the computational hydrogen electrode (CHE) model for reaction free energies and the Butler–Volmer equation for the potential dependence of the activation free energy (approximating the standard equilibrium activation voltage and the symmetry factor). They calculated reaction energies and barriers of a large reaction network where they identified a “low potential” (less reducing) ethylene and ethanol pathway via *CHO and a “high potential” (very reducing) pathway for methane and ethylene via *COH.^17^ It is not clear however how the low and high potential categorization was made, given that the rate-limiting barriers of both pathways are similar in magnitude (they found that the formation of *CHO is favored over *COH with activation energies of 0.64 vs 0.92 eV using the CHE and DFT-PBE). Furthermore, their calculated barriers suggest that formation of *CHO and its dimerization product OHC*—*CHO will kinetically overwhelm any other pathways for conversion of *CO, which ought to shut off formation of methane. The same authors predicted a high barrier for *CO coupling with and without two explicit solvating water molecules within DFT-PBE but focused on a rather strange-looking intermediate, O=C—*CO*, as proposed earlier by Calle-Vallejo and Koper.^15^

Goodpaster et al. used a self-consistent field constant electrode potential (CEP) DFT approach with the revised PBE (RPBE) XC functional (DFT-RPBE) and a Poisson–Boltzmann model for implicit solvation^25,26^ and charge compensation, to simulate formation of OC*–*CO and OHC–*CO on Cu(100) at different negative applied potentials^18^ (the authors only considered *CHO formation and coupling because of the work by Cheng et al. on C_1_ products that calculated a free energy barrier of formation from *CO of 0.55 and 1.45 eV for *CHO and *COH, respectively, from constrained molecular dynamics with DFT-PBE and arguably very short sampling trajectories of 2.4 ps per constrained structure).^27^ They found that, even without explicit water molecules, two *COs (at adjacent bridge sites, similar to Montoya et al.’s model^16^) may couple to form OC*–*CO but with a barrier that rather counterintuitively increased by ∼0.1 eV from ∼0.6 to 0.7 eV when going from 0.0 to −1.0 V vs RHE. The reaction free energy, however, went down from ∼0.5 to 0.2 eV at 0.0 to −1.0 V vs RHE. The reaction free energy lowering at negative potentials is consistent with Montoya et al.’s findings when they introduced an excess electron and a charge-compensating cation.^16^ It is clear that, despite the strangely opposite influence of applied potential on activation and reaction free energies in Goodpaster et al.’s simulations, OC*–*CO formation is weakly potential-dependent with implied electron transfer of ∼0.1 loss and ∼0.3 electron gain at the transition state and product, respectively. Because of this positive correlation between the barrier and magnitude of applied negative potential, the authors conclude that at large negative potentials, OC*–*CO becomes inhibited, where the C–C bond formation occurs through OHC–*CO.^18^

Garza et al. later investigated formation pathways for a wide range of C_2_ products, namely, ethylene, ethanol, acetic acid, acetaldehyde, ethylene glycol, and glycolaldehyde,^20^ also using DFT-RPBE and evaluating the potential dependence of free energies by applying both the CHE (for 0.0 vs RHE, same as Luo et al.’s approach)^17^ and Goodpaster et al.’s CEP method^18^ for potentials <0.0 V vs RHE. Garza et al. concluded that C–C formation on Cu(100) occurs via either OC*–*CO or OHC–*CO, where they form respectively at low and high (negative) potentials.^20^ Nonetheless, the former converts to the latter and is more thermodynamically favored than the conversion of *CO to *CHO. Therefore, the authors ascribe ethylene production to OHC–*CO.^20^

Molecular dynamics simulations from Cheng et al.^19^ (using DFT-RPBE and the D3 van der Waals correction to calculate atomic forces) and Kristoffersen and Chan^21^ (DFT-PBE-D3) led to similar conclusions as the static models, namely that OC*–*CO coupling on Cu(100) is kinetically feasible. Both bodies of work included explicit liquid water and a self-ionizing atom (Na in the former and Cs in the latter) leading to an excess electron in the Cu slabs in their models. Using metadynamics with the C–C distance designated as the collective variable, Cheng et al.^19^ and Kristoffersen and Chan^21^ calculated surmountable OC*–*CO coupling free energies of activation (at constant number of electrons) of 0.69 and 0.65–0.66 eV at room temperature, respectively.

All the above-mentioned computational studies use DFT, where many XC functional approximations incorrectly predict *CO to preferentially bind to hollow sites as opposed to the experimentally observed atop sites.^28–30^ This error can be ascribed to both XC delocalization error and a possibly too low CO π* energy, both of which favor artificially large back-bonding interactions from the metal to the molecule, which are enhanced at high coordination sites, e.g., hollow sites. The above DFT studies generally predicted facile *CO coupling with another *CO or *CHO on Cu(100), especially when considering solvation with explicit water molecules and the presence of excess electrons at the electrode surface. This coupling ability of *CO on Cu(100) is at the crux of current understanding of the unique activity of Cu to produce C_2_ products during ECO/CO_2_R.^2^

Wavefunction methods, when able to include important static and dynamic electron correlations, provide accurate electronic structure and energies. For example, Sharifzadeh et al. demonstrated that when using embedded multireference singles and doubles configuration interaction,^31^ they obtained the correct atop adsorption preference of CO on Cu(111), unlike pure DFT approximations.^32^ We previously investigated the one-proton–one-electron reduction of *CO using the embedded complete active space self-consistent field^33,34^ (ECASSCF) method followed by second-order perturbation theory^35–37^ (ECASPT2), enabled by density functional embedding theory (DFET),^38^ and showed that the formation of *COH is kinetically favored over *CHO via PCET on Cu(100).^39^ This behavior is unlike on Cu(111),^40^ which forms both with equal kinetic barriers. Furthermore, in the same study^39^ we calculated the relative stability of the different adsorption sites for *CO, *COH, and *CHO, also within ECASPT2. We found that *CO, *COH, and *CHO respectively favor atop (as expected), hollow, and bidentate-atop (i.e., both C and O are bound atop to two neighboring Cu atoms) adsorption configurations. For *CO, the bridge and hollow sites are 0.31 and 0.35 eV higher in energy than atop, implying that *CO can easily diffuse on Cu(100), with a modest barrier of around 0.3 eV. Both *COH and *CHO have higher diffusion barriers. If *COH is to migrate between hollow sites, it will go through bridge sites (as transition states) and *COH at a bridge site is 0.85 eV higher in energy than the hollow site.^39^ As for *CHO, it will likely diffuse between bidentate-atop sites via the C atom moving to a bridge site, which is 0.49–0.51 eV higher in energy than the bidentate-atop configuration.^39^ Although *CHO would be more mobile than *COH on Cu(100), the barrier to form *COH is ∼0.5 eV lower than *CHO at the relevant cathodic potential ranges of −0.5 to −1 V vs RHE.^40^ Based on these prior computational studies, here we explore the coupling of two *COs and a *CO with a *COH (given the high mobility of *CO) to determine the corresponding barrier to form precursors for C_2_ products early in the reaction on Cu(100), i.e., prior to multiple hydrogenation steps. Despite the high barrier for *COH diffusion, we also investigated the barrier for a pair of *COHs at adjacent hollow sites to couple, which would be relevant at potentials where all *COs are converted to *COHs at high coverages. We performed these simulations using DFT to determine structures and initial energetics that are subsequently refined with the more accurate ECASPT2, as we have done in our prior studies of ECO/CO_2_R.^39–41^

## Methods

### Modeling Overview

To calculate internal energies (electronic and nuclear contributions), we utilized embedded correlated wavefunction (ECW) theory, which involves multiple steps and different levels of theory. Specifically, we (1) optimized structures and reaction paths within periodic DFT with the Perdew–Burke–Ernzerhof (PBE) exchange-correlation (XC) functional^42^ and D3 van der Waals correction with Becke–Johnson damping (D3BJ),^43^ using slab Cu(100) models; (2) determined an effective cluster-environment interaction potential, i.e., embedding potential *V*_emb_), for a set of predetermined Cu clusters carved out from a pristine Cu(100) slab, via DFET;^38,44^ (3) from (1) we placed the structurally optimized adsorbates on the (structurally fixed) Cu clusters and re-evaluated the cluster + adsorbate energies within DFT and a CW method (vide infra) subject to *V*_emb_; and (4) we used the difference between the embedded cluster energies calculated from CW theory and DFT as a correction to the full periodic slab DFT energy. Below we provide the theoretical basis of the computational scheme and further details of the computational parameters used at each step.

### Periodic Slab DFT Calculations and Atomic-Scale Models

We performed periodic Kohn–Sham DFT calculations with the PBE XC functional within Vienna Ab-Initio Simulation Package (VASP)^45,46^ version 6.4.1 compiled with the VTST and VASP-sol subroutines. We used the projector augmented-wave (PAW) method^47^ and standard PAW potentials to represent the nuclei and frozen core electrons of H, C, O, and Cu, with self-consistently optimized 1*s*, (2*s*,2*p*), (2*s*,2*p*), and (4*s*,3*d*) valence electrons, respectively. We exclusively performed spin-restricted DFT, except to determine whether an adsorbate would yield radical character if allowed to do so, in which case we performed a spin-unrestricted DFT (specifically, for OC*–*COH, we tested both spin-polarized and unpolarized DFT, and found that despite having an odd number of electrons, this species prefers a spin-unpolarized solution when adsorbed on Cu). We used a planewave basis set with a kinetic energy cutoff of 660 eV. To aid electronic convergence with finite *k*-point sampling (vide infra), we smeared the occupation of the electronic states near the Fermi level using the Methfessel–Paxton scheme^48^ with a smearing width of 0.09 eV.

To simulate reactions on Cu(100) surfaces, we used periodic slab models with the repeated periodic images normal to the surface separated using ∼15 Å of space, which is either vacuum or is filled with a dielectric continuum representing liquid water. We used the optimized lattice constant of face-centered cubic Cu (conventional cubic cell constant *a* = 3.567 Å) from ref 49 that used similar computational parameters as we have here. We utilized a 100-atom, four-layer (5 × 5) supercell Cu(100) slab, with adsorbates placed on only one surface and the two layers comprising the other surface and subsurface held fixed to their bulk positions (Figure 1, top panels) to mimic a semi-infinite bulk crystal. The periodic cell’s surface normal vector length is 25 Å with in-plane lattice constants of 12.611 Å × 12.611 Å. All three lattice vectors are orthogonal. Accordingly, we used a 4 × 4 × 1 Monkhorst–Pack *k*-point mesh^50^ to sample the Brillouin zone for an in-plane reciprocal lattice spacing of ∼0.02 2πÅ^–1^.

**Figure 1:**
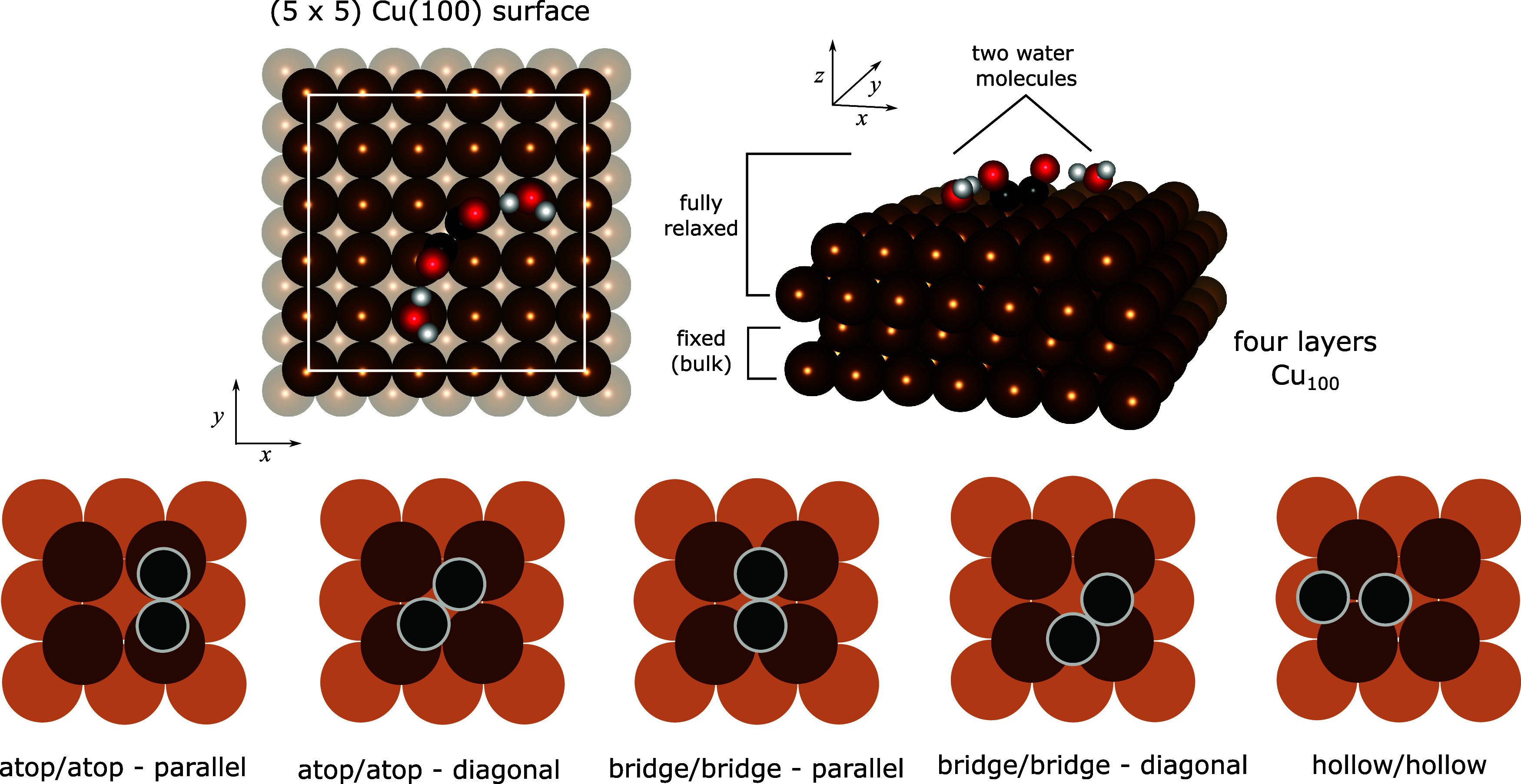
Top, simulation supercell (left: top view, right: side view) showing a four-layer (5 × 5) supercell Cu(100) laterally periodic slab. OC*–*CO with C atoms attached to the atop sites of the surface and two solvating water molecules are shown as an example. Cu - dark brown spheres, C - black, O - red, and H - white. Vectors: *x* = [011], *y* = [011], *z* = [100]. Bottom, schematic of sampled initial adsorption configurations with C atoms as attachment points of the *CO and *COH coupling products on Cu(100). Surface Cu - dark brown, subsurface Cu - light brown, and C - black.

We first performed simulations in vacuum to determine the favorable adsorption configurations for the coupling of two *COs, a *CO and a *COH, and two *COHs, along with two adsorbed water molecules hydrogen-bonded to the O atoms. Literature shows that adsorbed waters help stabilize OC*–*CO.^16^ For consistency, we introduced two adsorbed waters hydrogen-bonded to the O atoms of all coupling products (with H_2_O as hydrogen-bond donor). We sampled different adsorption sites for the two C atoms of the coupling products, where we considered atop, bridge, and hollow sites for each C atom (Figure 1, bottom panels). We then placed the adsorbed waters onto adjacent atop Cu sites to enable hydrogen bonding. Not all adsorption configurations remained in their initial guess positions. Figures S1–S3 show the optimized adsorption configurations for OC*–*CO, OC*–*COH, and HOC*–*COH, respectively. We report therein only the structures that are unique, either because of the adsorption sites for the C atoms or the arrangement of the solvating water molecules relative to the coupling products. Tables S1–S3 summarize the relative energies in vacuum of the structures in Figures S1–S3.

We optimized the atoms in the slabs while fixing the lattice constants and freezing the bottom two layers of the slab to their equilibrium bulk positions. We relaxed all structures until the maximum absolute atomic force of 0.03 eV/Å is reached for all the atoms allowed to move. We use a looser threshold than the typical value of 0.01 eV/Å because DFT-PBE+D3BJ heavily favors both the bridge and hollow site adsorption of *CO and the atop configuration is a shallow minimum, and thus the tighter convergence threshold on forces may eventually lead to relaxation of *CO to either bridge or hollow site.

We determined the minimum-energy reaction path, via the climbing-image nudged elastic band (CI-NEB) method,^51^ for forming the most favored adsorption configurations as determined within ECW theory (Tables S1–S3, vide infra) both in vacuum and in dielectric continuum water (Poisson–Boltzmann model for implicit solvation),^25,26^ with the structures obtained from the former used as initial guesses for the latter. We used a force constant of 5 eV/Å^2^ for the fictitious spring linking images along the reaction coordinate.^51^ We set a higher force threshold of 0.05 eV/Å for the convergence of all of the NEB images, due to the higher dimensionality of the optimization space to balance computational cost and accuracy.

### Free Energy Evaluation

We calculated the vibrational free energy contributions from the atoms of the adsorbate and 12 select topmost Cu surface layer atoms (Figure S4) by numerically evaluating the (partial) Hessian. Partial Hessians were constructed via finite displacements of the atoms by ±0.015 Å along the three Cartesian directions and evaluating the resulting change in the atomic forces. From these vibrational frequencies we confirm that all the stationary structures (reactants and products) chosen for the coupling reactions and transition states are minima and first-order saddle points, respectively.

We calculated the adsorbate/surface enthalpies and free energies using statistical mechanics within the harmonic-vibration approximation.^52^ For all the relevant stationary structures, we found no imaginary vibrational frequencies with magnitude greater than 100*i* cm^–1^. Transition states, i.e., first-order saddle points, only have one vibrational frequency >100*i* cm^–1^. In evaluating the vibration free energy contributions, we used a minimum threshold of 100 cm^–1^ for the real-valued vibrational modes. The vibrational Helmholtz free energy *A*_vib_) at temperature *T* of the surface is given by

1 $$A_{vib}\left(T\right)=\underset{i}{\sum }\frac{hv_{i}}{2}+k_{B}T\;\ln\left(1-e^{-hv_{i}/k_{B}T}\right)$$

The summation above is for all (nontrivial) modes with (real) vibrational frequencies (*v*_*i*_) > 100 cm^–1^ as described above. *h* and *k*_B_ are Planck and Boltzmann constants, respectively. The first term, $\frac{hv_{i}}{2}$ , is the zero-point energy (ZPE).

### Partitioning Scheme and Embedding Potential Optimization

We carved out Cu_18_ and Cu_17_ clusters from a pristine, 100-atom, four-layer (5 × 5) supercell slab to represent the active site, where we include six second-layer subsurface Cu atoms and 12 or 11 surface Cu atoms to create the clusters (Figure S5). We thus designate the remaining 82 or 83 Cu atoms as the environment (Figure S5). We used the Cu_18_ and Cu_17_ clusters for adsorbates with even and odd number of electrons, respectively, to maintain an overall even number of electrons. An even total number of electrons ensures nonmagnetic cluster models, the same as the slab models. We also chose cluster sizes and shapes that explicitly include all direct interactions of the adsorbates with the surface for all structures along the reaction path, rendering all such interactions to be described fully quantum mechanically. As we have shown previously, the energies are insensitive to cluster size if so.^53^

To derive the interaction of the clusters with their respective environments, we employed DFET^38^ where we solve for an optimized effective (local) potential by using an extended Wu–Yang functional.^54^ The unique optimized effective potential (OEP), which we refer to as the embedding potential or *V*_emb_, serves to embed the fragments (here the cluster and its environment) and mutually acts on the fragments. Ultimately, the *V*_emb_ convergence criterion is that the sum of the fragments’ self-consistent electron densities determined in the presence of *V*_emb_ reproduces the full system self-consistent electron density, within DFT. To do so in DFET, a scalar quantity *W* is maximized (or equivalently −*W* is minimized) instead of directly minimizing the density residual (a three-dimensional array)

2 $$-W=\left\{\int V_{emb}\rho^{full}\left(r\right)dr\right\}-E_{eDFT}^{cl}\left[\rho^{cl}\left(r\right),V_{emb}\right]-E_{eDFT}^{env}\left[\rho^{env}\left(r\right),V_{emb}\right]$$

where cl and env denote the cluster and environment fragments of the full system, to determine *V*_emb_ *. ρ* ^*i*^ and *E*_eDFT_^*i*^ are the self-consistent electron density and energy of *i* = cluster, environment, or full system from DFT. *V*_emb_ is added to the one-electron Hamiltonian of the fragments *H*_elec_^°^) to yield the embedded Hamiltonian: *H*_elec_^emb^ = *H*_elec_^°^ + *V*_emb_. The functional derivative of eq 2 with respect to *V*_emb_ is

3 $$-\frac{\delta W}{\delta V_{emb}\left(r\right)}=\rho^{full}\left(r\right)-\rho^{cl}\left(r\right)-\rho^{env}\left(r\right)$$

which in practice is evaluated as

4 $$-\frac{\delta W}{\delta V_{emb}\left(r\right)}=\frac{\delta E_{DFT}^{full}}{\delta V_{emb}\left(r\right)}-\left(\frac{\delta E_{eDFT}^{cl}}{\delta V_{emb}\left(r\right)}+\frac{\delta E_{eDFT}^{env}}{\delta V_{emb}\left(r\right)}\right)$$

Therefore, at the minimum of −*W*

5 $$\rho^{full}\left(r\right)=\rho^{cl}\left(r\right)+\rho^{env}\left(r\right)$$

We optimize these *V*_emb_ values using VASP^55^ v. 6.4.1 compiled with the PAW DFET subroutine^55,56^ along with the standard limited-memory Broyden–Fletcher–Goldfarb–Shanno with bound constraints (L-BFGS-B) minimization scheme implemented in the scipy.optimize.fmin_l_bfgs_b routine available in python. We generate the *V*_emb_(*r*) on a Cartesian real-space grid. Figure S5 shows the optimized *V*_emb_(*r*) values for Cu_18_ and Cu_17_ clusters from a pristine four-layer (5 × 5) Cu(100) slab, which we used in the ECW calculations described below.

### ECW Theory Calculations

We performed all ECW theory calculations within Molpro v. 2024.^57^ We used atom-centered Gaussian-type basis sets, namely, the all-electron correlation-consistent polarizable valence double-ζ (cc-pvdz)^58^ for H, C, and O; we used the LANL2DZ^59^ basis set and accompanying 10-electron-core effective core potential for Cu.^60^ Accordingly, we transformed *V*_emb_ from Cartesian grid representation to Gaussian basis representation using our group’s embedding integral generator code^61^ prior to its addition to the one-electron Hamiltonian.

In our ECASSCF calculations, we determine the size and identity of the AS by considering the number of valence electrons of the adsorbates and bonding interactions they form with the Cu surface (see Supplementary Methods and Figures S6–S12). We employed active space (AS) sizes of 14 electrons in 14 orbitals ((14e,14o)) and (12e,12o), respectively for OC*–*CO and OC*–*COH to evaluate static correlation. For HOC*–*COH, we used ASs of (10e,10o) and (12e,12o) for determining its preferred adsorption configuration and coupling energetics calculations, respectively. We used a larger AS for the latter because the reactant, i.e., the two monomeric *COHs, forms more bonds with Cu (three for each *COH, treated with a (6e,6o) AS) than their coupling product (unlike in the other two species). Correlation consistency then demands a (12e,12o) AS for the entire reaction path, while this is not necessary for the adsorption energetics. We employ a combination of “creeping” and “orbital merging” to generate initial guess orbitals,^53^ which we found to be an effective method for obtaining a consistent active space. As we have done in refs 39–41 we followed CASSCF with CASPT2 to evaluate dynamic correlation contributions of all other one- and two-electron excitations not captured in CASSCF except for excitations involving the core orbitals, namely, orbitals derived from the core 1*s* states of the C and O atoms, and outer-core 3*s* and 3*p* states of the Cu atoms. We do not expect core electron correlations will contribute significantly to the reaction energies (energy differences). For CASPT2, we used an ionization potential-electron affinity (IP-EA) shift^62^ of 0.25 Ha and level-shift corrections^63^ of 0.20 and 0.30 Ha for the Cu_18_ and Cu_17_ clusters, respectively. The Cu_17_ clusters with adsorbates required the higher level-shift value to have all the images along the reaction path avoid PT2 energy divergence. We performed a convergence test for the OC*–*CO coupling reaction using an AS of (10e,10o) by removing two pairs of occupied and virtual orbitals that are most and least populated from (14e,14o), respectively (Figure S6 and S10), as such orbitals represent the least correlated electrons in the AS. We down-sampled from (14e,14o) because it is already the largest AS we can viably perform. We used (10e,10o) because it retains the symmetric representation of the two *COs, whereas (12e,12o) does not. Figure S13 shows that the effective activation energy remains the same when going from AS­(10e,10o) to AS­(14e,14o). We expect the same or better convergence error for our AS (14e,14o) simulation when compared to larger ASs, because the larger AS already accommodates all possible Cu–CO and OC–CO valence electron interactions. Importantly, the convergence errors we found in Figure S13 are far less than the difference between DFT-PBE+D3BJ and ECASPT2 predictions, which is ∼1 eV (vide infra).

To calculate the total energy within ECASPT2, we express the full system energy (*E*_ECASPT2_^full^) as

6 $$E_{ECASPT2}^{full}=E_{DFT‐PBE+D3BJ}^{full}-E_{D3BJ}^{full}+\left(E_{eCASPT2}^{cl}\left[V_{emb}\left(r\right)\right]-E_{eDFT‐PBE}^{cl}\left[V_{emb}\left(r\right)\right]\right)$$

where *E*_eCASPT2_^cl^[*V*_emb_] and *E*_eDFT‑PBE_^cl^[*V*_emb_] are respectively the (nonperiodic) embedded cluster (cl) energies within CASPT2 and DFT-PBE using the Gaussian-type basis sets (vide supra) calculated using Molpro. *E*_DFT‑PBE+D3BJ_^full^ and *E*_D3BJ_^full^ are respectively the total DFT-PBE+D3BJ energy and the D3BJ energy only of the full periodic slab with the adsorbate calculated within VASP (vide supra). We removed *E*_D3BJ_^full^ from the total energy because *E*_eCASPT2_^cl^[*V*_emb_] should already contain the correct medium-to-long-range van der Waals interaction. By removing D3BJ from both the slab and cluster DFT calculations and simply incorporating the ab initio van der Waals interactions in the active region, we reduce empiricism in the total energy. Here, both *E*_eCASPT2_^cl^[*V*_emb_] and *E*_eDFT‑PBE_^cl^[*V*_emb_] were calculated in a vacuum. The CASPT2 correction therefore is applied only for vacuum electronic structure. *E*_DFT_^full^, on the other hand, is calculated either in vacuum (for structures optimized in vacuum) or in implicit water via the VASP polarizable continuum model (for structures optimized in implicit water). The proper solvation free energy therefore is captured already within DFT; thus, the global solvation energy calculated for the full system (vide supra) need not be regionally corrected within the ECW framework as a first-order approximation. A technical reason for this approximation is the incompatibility of *V*_emb_(*r*) with implicit solvation models for isolated clusters, where the dielectric screening effects due to the solvent would also apply to the cluster sides that would not otherwise interact with the solution had it been embedded within a slab.

## Results

### Coupling Structures

We begin by screening for the preferred adsorption configurations of OC*–*CO, OC*–*COH, and HOC*–*COH within the final of level of theory, i.e., ECASPT2. This screening procedure entails sampling different adsorption configurations for these species (Figure 1) and structurally optimizing them within DFT-PBE+D3BJ. Figures S1–S3 show the optimized candidate structures for OC*–*CO, OC*–*COH, and HOC*–*COH, respectively. Tables S1–S3 summarize their relative energies within DFT-PBE+D3BJ and ECASPT2. We use representative gas-phase C_2_ hydrocarbons and oxygenates calculated within the same level of theory (Table S4) as references for identifying C–C and C–O bond orders using bond lengths as a metric.

For OC*—*CO, among the multitude of possibilities, the resulting C—C and C=O bond lengths suggest that all stable configurations found have C—C single bonds (compare the Lewis structures in Figure S1 with Table S4) with zero formal charge, optimizing to either atop/atop or bridge/bridge configurations only. The atop/atop configuration exhibits two distinct motifs, one with the C—C bond axis is parallel to one of the sides of the square Cu surface lattice unit cell and the other in which it is diagonal to it; these are denoted accordingly as atop/atop – parallel and atop/atop – diagonal. For bridge/bridge, the only stable structure has the bridge sites at the opposite ends of a square Cu surface lattice unit cell, i.e., bridge/bridge – parallel (Figure S1). DFT-PBE+D3BJ predicts the bridge/bridge – parallel configuration to be the most favorable (as already reported),^16,18,19^ while the atop/atop configurations are 0.23 and 0.43 eV higher in energy. In stark contrast, ECASPT2 flips the order, predicting atop/atop – parallel to have the lowest energy among the three, with both other structures ∼0.45 eV less stable (Table S1). Later, we will show the energetics for the formation of the atop/atop – parallel configuration (lowest ECASPT2 energy), as well as the bridge/bridge – parallel configuration (prominently featured in the computational literature) for comparison.

For OC*–*COH, despite sampling different initial adsorption sites for the C atoms, all structures converged to bridge/bridge-type configurations with four unique structures. These structures are differentiated by both the presence (or absence) of internal hydrogen bonding between the COH end to the CO end of the molecule and by the relative positions of the two water molecules that hydrogen-bond with them (Figure S2). The three lowest energy structures feature intramolecular hydrogen bonding and differ in energy only by ∼0.1 eV (Table S2). Given the energies at this stage are evaluated in vacuum, we caution against overemphasis of internal hydrogen bonding because solvation should stabilize the highest energy structure without internal hydrogen bonding. We assign OC*–*COH a partial negative charge because the C–C and carbonyl C–O bond lengths are between a double and a single bond, i.e., 1.5 bonds (compare the bond lengths in Figure S2 with Table S4). A resonance structure where the double bond alternates between the C–C and carbonyl C–O bonds can explain such a structure. For the singly bonded C–O, O must have a negative formal charge. Figure 2 shows the proposed resonance structure, and since the singly bonded C–O is only present in one of the resonance structures, OC*–*COH must be only partially anionic δ−). Henceforth, we will interchangeably refer to OC*–*COH as [OC*–*COH]^δ−^. The partial anionic charge on OC*–*COH also means that the four Cu atoms surrounding the adsorbate must carry a partial positive charge for charge balance on a neutral Cu surface corresponding to zero applied potential. Hereafter, we refer to these atoms collectively as Cu_4_ or Cu_4_^δ+^, when respectively referring to the neutral or partial positively charged state. Note that if a negative potential were applied as in an actual electrocatalytic reduction setup, this partly negatively charged species would be even more likely to form, and without positive charge compensation at the surface.

**Figure 2:**
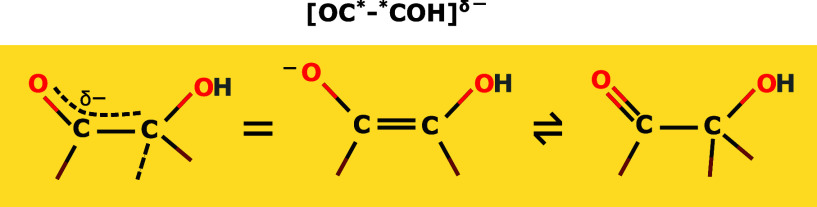
Bonding resonances that illustrate emergence of partial negative charge on OC*–*COH surface species (average of the two resonance structures shown on the left).

We only re-evaluated the energy within ECASPT2 for the two lowest-energy structures within DFT-PBE+D3BJ and found them to have essentially the same energy (Table S2). With ECASPT2, differences in stabilization due the proximity of the explicit water molecules is less impactful. Within DFT-PBE, especially with D3BJ corrections, hydrogen-bonding is known to be overly favored,^43^ which may explain the sensitivity of the energy to the water configuration within DFT-PBE+D3BJ. For *CO and *COH coupling, we investigated only the formation of the [OC*–*COH]^δ−^ configuration that is lowest in energy within DFT-PBE+D3BJ, because ECASPT2 predicts the two structures that have lowest energy in DFT-PBE+D3BJ to be nearly degenerate (within 0.01 eV) anyway.

Finaly for HOC*–*COH, we found three nearly identical bridge/bridge structures that feature internal hydrogen bonding similar to [OC*–*COH]^δ−^ (Figure S3). We also managed to stabilize an atop/atop – diagonal structure by positioning two water molecules in a way that blocks the rotation of HOC*–*COH that may lead to its relaxation to bridge/bridge configuration (Figure S3). All C–C bonds exhibit extended double-bond character, like in an ethendiol (compare the Lewis structures and annotated bond lengths in Figure S3 with Table S4). DFT-PBE+D3BJ predicts all bridge/bridge configurations to be favorable and nearly degenerate, with atop/atop – diagonal 0.3 eV higher in energy (Table S3). We recalculated the relative energy of the lowest-energy bridge/bridge and atop/atop – diagonal configurations and found that ECASPT2 and DFT-PBE+D3BJ qualitatively and nearly quantitatively agree, with atop/atop – diagonal 0.2 eV higher in energy. We therefore calculated only the coupling of two *COHs to form the bridge/bridge configuration, as we describe below.

### Coupling Pathways

We considered for completeness the formation of two OC*–*CO configurations, namely, atop/atop – parallel and bridge/bridge for comparison, despite the latter being substantially higher in energy within ECASPT2 (but is the ground state within DFT-PBE+D3BJ). Figure 3A displays (left to right) the reaction path for the former, preferred product, in vacuum. Starting from two atop *COs (atop + atop) that are separated by ∼3.6 Å (second-nearest-neighbor atop sites), one of the *COs diffuses toward an atop site nearest to the other via a bridge site (a second atop + atop configuration). The two C atoms then drift toward each other, while the O atoms tilt away from each other, until an optimal single C–C bond length is achieved (1.60 Å) at the product state (atop/atop – parallel). DFT-PBE+D3BJ predicts a transition state (TS) with C–C bond length of 1.84 Å. The C–O bonds lengthen from 1.16 to 1.20 Å at the TS, and finally to 1.23 Å at the product. Figure S14 shows the corresponding pathway for the bridge/bridge – parallel from a similar atop + atop configuration.

**Figure 3:**
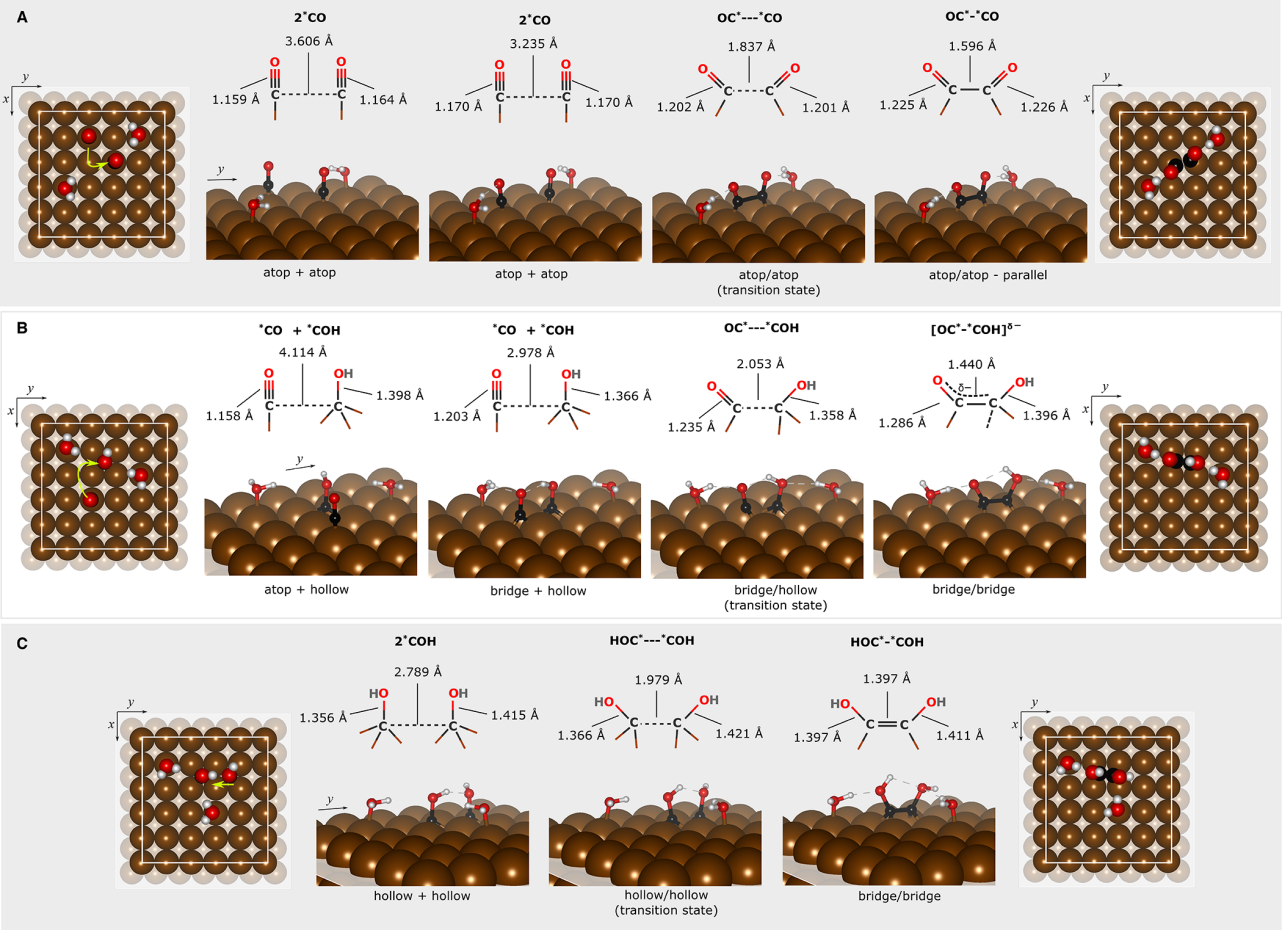
DFT-PBE+D3BJ vacuum-optimized structures along the minimum-energy coupling paths for the formation of the most ECASPT2-stable (A) atop/atop – parallel OC*–*CO, (B) bridge/bridge OC*–*COH, and (C) bridge/bridge HOC*–*COH configurations. The two (top and side views) leftmost and two (side and top views) rightmost panels for each row are the reactant and product. The structures in between the reactants and products are either the transition state (as marked) or a local minimum. The structures are labeled according to the adsorption site of the *COs and/or *COHs. If the species are uncoupled or coupled, the label shows “+” or “/”, respectively. The structures shown here correspond to the data points marked with red circles in their respective reaction energy curves in Figure 4A. For the top views, the white boxes show the boundary of the periodic (5 × 5) supercells, and on the reactant, the neon green arrows chart schematically the trajectory of the molecules as they form the product. The C–C and C–O distances are annotated in their corresponding Lewis structures. Cu - dark brown spheres, C - black, O - red, and H - white. Vectors: *x* = [011], *y* = [011], *z* = [100].

The reactant for the formation of [OC*–*COH]^δ−^ has the *COH at a hollow site and a *CO atop one of the second-nearest-neighbor Cu to *COH (Figure 3B). We placed the *COH such that the pathway will not require the *COH to move too far because the diffusion energy for *COH is high, e.g., calculated to be ∼0.9 eV via the bridge site,^39^ whereas *CO is mobile on pristine Cu(100).^39,64^ Atop *CO moves through a hollow site to a bridge site, where now the *CO shares a common Cu atom with *COH. At this point, the *CO remains upright. During this step, the hydroxyl of *COH also rotates around the C–O axis such that it forms a hydrogen bond with the carbonyl oxygen. This structure is a local minimum (vide infra) where one of the water molecules and the *COH hydrogen bond with *CO (Figure 3B). This step is followed by *CO diffusion to another bridge site via a hollow site, where this time *CO shares two Cu atoms with *COH. As the C atom of *CO moves closer to *COH, the *COH is pushed toward the opposite bridge site, while the carbonyl and hydroxyl O atoms tilt away from each other until [OC*–*COH]^δ−^ forms in a bridge/bridge configuration and the carbonyl C–O bond lengthens (Figure 3B).

Coupling of two *COHs on adjacent hollow sites to form HOC*–*COH begins with one of the *COHs moving over to the bridge shared by the two adsorbates (Figure 3C). As one of the *COHs moves, the interaction of the two species is repulsive, causing the other *COH to be pushed away to the other bridge site. The presence of a water molecule at a nearby atop site, along with the fictitious NEB spring, help prevent this *COH from simply diffusing into the next hollow site. As the two *COHs gets closer, a double bond forms with the C atoms now residing at their respective bridge sites.

### Coupling Energetics

Figure 4A shows the potential energy curves (PECs) calculated in vacuum from DFT-PBE+D3BJ (gray) and ECASPT2 (light blue) for the formation of atop/atop – parallel and bridge/bridge OC*–*CO (first and second row, first column), [OC*–*COH]^δ−^ (first row, second column), and HOC*–*COH (first row, third column). The ECASPT2 curves use the same structures as in DFT-PBE+D3BJ and therefore have data points at the same reduced reaction coordinates (*R*_C_). We define *R*_C_ as the cumulative length (i.e., a one-dimensional representation) of the path in 3*N*_a_-dimensional space (*N*_a_ = total number of atoms in the simulation) defined by the CI-NEB images (vide supra) starting from the reactant (*R*_C_ = 0) and terminating at the product. We normalized *R*_C_ by the total path length to obtain the dimensionless reduced *R*_C_, which is equal to one at the product. Table S5 summarizes the internal energies of reaction and activation from both DFT-PBE+D3BJ and ECASPT2 (except for bridge/bridge OC*–*CO, which is highly disfavored, vide supra).

**Figure 4:**
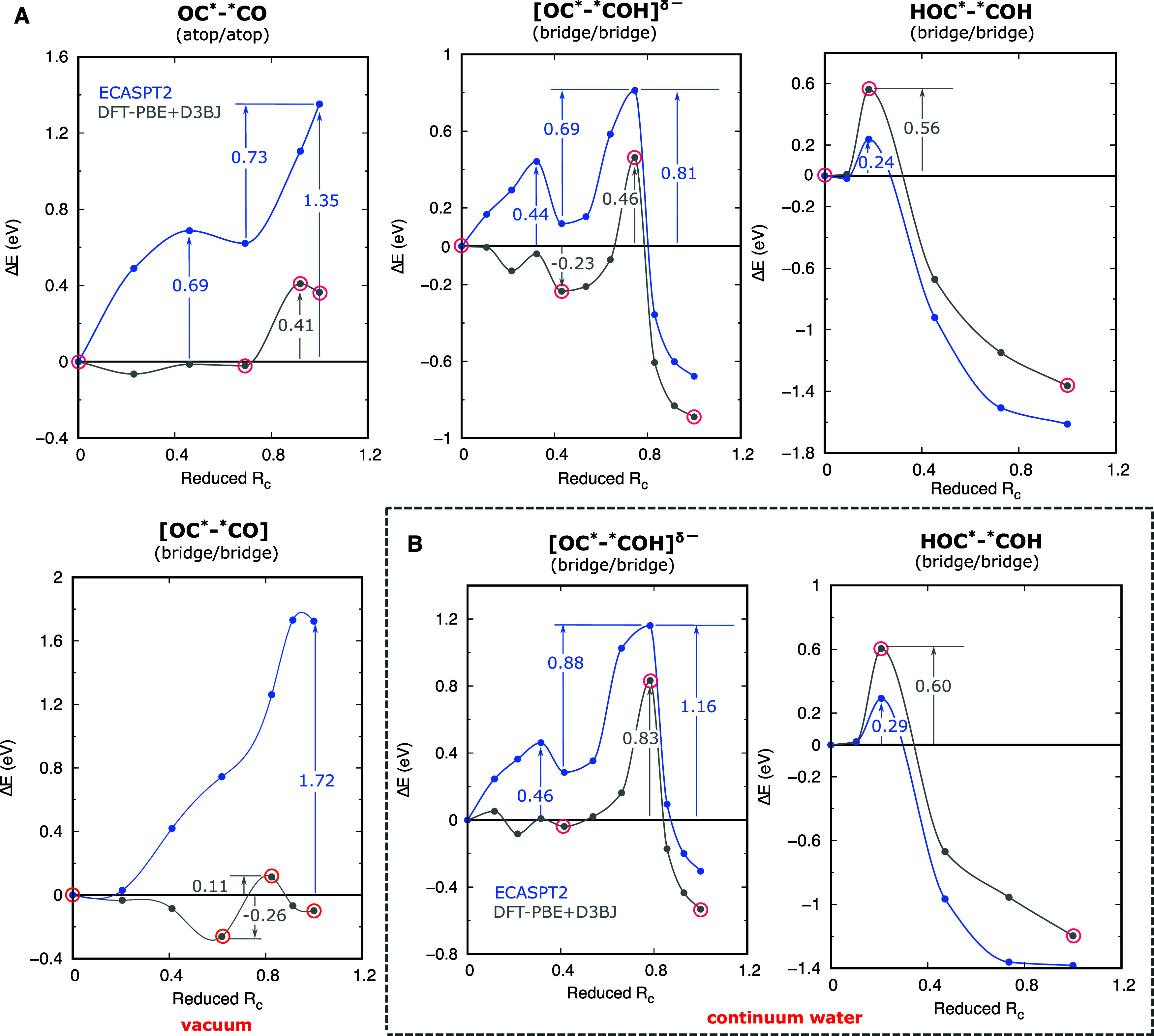
C–C coupling energy curves. (A) DFT-D3BJ (gray) and ECASPT2 (light blue) reaction energy curves in a vacuum for atop/atop – parallel and bridge/bridge OC*–*CO, bridge/bridge OC*–*COH, and bridge/bridge HOC*–*COH configurations (as labeled). The corresponding structures for the data points marked with red circles are shown in Figures 3A–C, respectively, for the first row and Figure S14 for bridge/bridge OC*–*CO. (B) Corresponding reaction energy curves for bridge/bridge OC*–*COH and bridge/bridge HOC*–*COH configurations (as labeled) in continuum water. The corresponding structures for the data points marked with red circles are respectively shown in Figures 5B and 5C. For all plots, reaction barriers and some reaction energies are marked (numbers on the figures are all in eV).

For the formation of atop/atop OC*–*CO, as one of the *COs migrates to an atop site adjacent to the other (Figure 3A), the PEC is flat within DFT-PBE+D3BJ but is activated within ECASPT2 with a barrier of 0.69 eV at reduced *R*_C_ = 0.46. This indicates strong repulsion between the two *COs as they get closer in the latter level of theory (Figure 4A). The ECASPT2 PEC also shows a clear local minimum for when the *COs are at adjacent atop sites (reduced *R*_C_ = 0.69). As the C atoms form a bond, the energy increases by 0.73 eV from that local minimum. The effective energy barrier for the coupling is 1.35 eV, far higher than what DFT-PBE+D3BJ predicted (0.41 eV). On the other hand, for bridge/bridge OC*–*CO, we see a deeper local minimum (−0.26 eV) within DFT-PBE+D3BJ for when the two *COs are at bridge sites right before coupling (Figure S14) at reduced *R*_C_ = 0.62 (Figure 4A). There is then a modest barrier for coupling from this DFT-predicted minimum (0.11 + 0.26 = 0.37 eV) and slight endothermicity (0.16 eV). These energetics are consistent with Montoya et al.’s predicted activation and reaction energies of ∼0.4 and 0.3 eV, respectively, for a similar reaction pathway (also without excess charge) but with more explicit water molecules included (vide supra).^16^ ECASPT2 however, reveals a highly activated (high-barrier) coupling reaction, with a barrier (that is also the reaction energy) of 1.7 eV and without any local minimum at reduced *R*_C_ = 0.62.

The two-step diffusion of *CO toward a *COH to form [OC*–*COH]^δ−^ is clear in both DFT-PBE+D3BJ and ECASPT2 PECs, with a local minimum at reduced *R*_C_ = 0.43 (Figure 4A). The first leg of the reaction, diffusion of *CO from an atop site to a bridge site adjacent to *COH (Figure 3B), has a surmountable ECASPT2 barrier of 0.44 eV, which is only ∼0.1 eV higher than the diffusion barrier of *CO on pristine Cu(100).^39^ In the second step, as *CO encroaches into *COH’s adsorption site, the energy increases by 0.69 eV before it drastically drops to −0.68 eV within ECASPT2 (Figure 4A). The effective barrier is the sum of the reaction energy of the first step and the barrier of the second, which totals 0.81 eV within ECASPT2. DFT-PBE+D3BJ presents a qualitatively similar picture as CASPT2, except it predicts the *CO at the bridge site adjacent to *COH to be lower in energy than an atop *CO farther away from *COH and the reaction is more exothermic by −0.2 eV (Figure 4A).

In the formation of HOC*–*COH (Figure 3C), as the double bond forms between the two C atoms, the interaction between the two *COHs becomes more favorable within ECASPT2 (Figure 4A) suggesting that two adjacent *COHs should spontaneously form HOC*–*COH subject to a small barrier of 0.24 eV. DFT-PBE+D3BJ predicts a higher barrier of 0.56 eV. Furthermore, unlike in OC*–*CO and [OC*–*COH]^δ−^, ECASPT2 predicts a more exothermic reaction than DFT-PBE+D3BJ (by −0.25 eV) (Figure 4A). While this behavior within ECASPT2 seems unusual, if one looks at the ECASSCF active space natural orbitals for the transition state (Figure S12, right panel), there is already a clear formation of C–C σ-bonding-like character and the most correlated orbitals already exhibit a C–C π-bonding-like and antibonding-like symmetry with occupation numbers of 1.92 and 0.09 electrons. The manifestation of favorable bonding interactions between the monomers even before a bond is expected to form (1.98 Å vs ∼1.5 Å for a single C–C bond—Figure 3C and Table S4) signals early transition-state character (as expected for very exothermic reactions) and enables facile bond formation.

Table 1 summarizes corresponding reaction and activation free energies at room temperature (298.15 K) using reaction and activation energies without ZPE from DFT-PBE+D3BJ and ECASPT2 (Figure 4 and Table S5). A comparison of Table S5 and Table 1 reveals that finite temperature effects (eq 1) on the free energetics are small (within ∼0.1 eV), and thus the observations made above looking only at internal energies hold even within the context of free energetics at 298.15 K. Generally, finite temperature corrections yielded more positive (or essentially unchanged) barriers and lesser exothermicity (as in the case of [OC*–*COH]^δ−^ and HOC*–*COH). In the next section, we explore the effect of dielectric continuum implicit solvation with the dielectric constant of pure water, i.e., “continuum water” (Methods).

**Table 1:** Calculated Reaction and Activation Free Energetics in Vacuum and Continuum Water at 298.15 K^a^

	Δ*G*_rxn_ [eV]		*G* ^⧧^ [eV]	
Reaction^b^	DFT-PBE+D3BJ	Emb-CASPT2	DFT-PBE+D3BJ	Emb-CASPT2
Vacuum				
2*CO → OC*–*CO (atop/atop)	0.44	1.43	0.48	1.43^c^
*CO + *COH + Cu_4_ → [OC*–*COH]^δ−^ + Cu_4_^δ+^	–0.73	–0.52	0.52	0.87
2*COH → HOC*–*COH	–1.23	–1.47	0.55	0.23
Continuum Water (ε = 78.4)				
2*CO → OC*–*CO (atop/atop)	0.67	1.74	^d^	1.74^c^
*CO + *COH + Cu_4_ → [OC*–*COH]^δ−^ + Cu_4_^δ+^	–0.42	–0.19	0.86	1.19
2*COH → HOC*–*COH	–1.10	–1.29	0.59	0.27

a Table S5 summarizes the internal energies (w/o ZPE) used in conjunction with eq 1 to calculate free energies.

b We explicitly include Cu_4_^δ+^ in [OC*–*COH]^δ−^ for charge balance.

c The same as *ΔE*_rxn_.

d CI-NEB not performed.

### Implicit Solvation Effects

We also explored the effect of implicit solvation on the optimized structures and coupling free energetics. We rerelaxed the vacuum-optimized stationary structures in the dielectric screening of the water continuum model, except for the bridge/bridge OC*–*CO, which is significantly higher in energy than the atop/atop configuration in vacuum. We also performed the same reaction path optimization under solvation, but not including the pathway for atop/atop OC*–*CO, which we know from vacuum calculations to have no transition state and to be already highly endoergic. Figure 5 shows the results after optimization in continuum water for select structures. The figure shows the same set of structures that appear in Figure 3, except for the reactants. Comparing Figures 3 and 5, it is evident that the presence of the solvent has negligible influence on both the stationary and TS structures, with bond lengths changing less than ∼0.05 Å.

**Figure 5:**
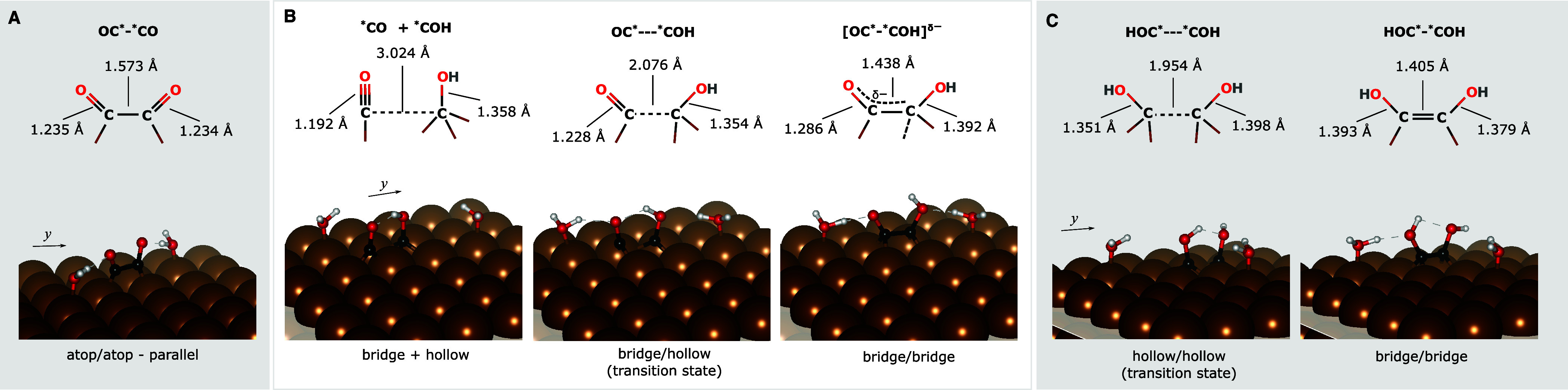
DFT-PBE+D3BJ solvent-optimized structures along the minimum-energy coupling paths (not including the reactant) for the formation of the most ECASPT2-stable (A) atop/atop – parallel OC*–*CO, (B) bridge/bridge OC*–*COH, and (C) bridge/bridge HOC*–*COH configurations. See Figures 3A–C for comparison, although here, only the transition states, a local minimum (for B), and products are shown. The structures are labeled according to the adsorption site of the *COs and/or *COHs. If the species are uncoupled or coupled, the label shows “+” or “/”, respectively. The structures shown in (B) and (C) correspond to the data points marked with red circles in their respective reaction energy curves in Figure 4B. The C–C and C–O distances are annotated in their corresponding Lewis structures. Cu - dark brown spheres, C - black, O - red, and H - white. Vectors: *x* = [011], *y* = [011], *z* = [100].

Figure 4B shows (as labeled) the re-evaluated potential energy curves for the formation of [OC*–*COH]^δ−^ and HOC*–*COH (see also Table S5). The figure shows that the influence of the solvent on the energetics is more dramatic than on the structures. The change in the effective barrier to the formation of [OC*–*COH]^δ−^ due to solvation is ∼0.35 eV both within DFT-PBE+D3BJ and ECASPT2. (Note that the solvation effect is the same for both levels of theory, since the solvation model in our scheme only acts on the DFT-PBE+D3BJ calculations.) The exothermicity of both reactions were also reduced by ∼0.2–0.4 eV. As for the formation of atop/atop OC*–*CO, the reaction becomes even more endothermic by ∼0.3 eV (Table S5). Table 1 also shows the corresponding free energetics under solvation, showing the same trend as in the internal energetics. Based on ECASPT2, the free-energy change associated with the formation of atop/atop OC*–*CO is now immense (1.74 eV) and the barrier for the formation of [OC*–*COH]^δ−^ is now more than 1 eV. Formation of HOC*–*COH, however, remains exoergic (although less so than in vacuum) and with a nearly unchanged small barrier.

The upward (destabilizing) effect of implicit solvation on both barriers and reaction energies, especially involving *CO, seems counterintuitive at first. After all, the products are more polar (and in some cases charged) than *CO and thus should energetically benefit the most from solvation. On the other hand, if one considers the interaction of the explicit water and C-based molecules, *CO (reactant) and H_2_O hardly interact, however as the product forms, stronger hydrogen bonding interactions between the explicit water and coupled products emerge. This is also true for HOC*–*COH because we placed the water molecules so that they optimally interact. In the reactant state, however, the *COH and H_2_O are farther away from each other (Figures 3C and 5C, left panel), and we did ensure that the water molecules stay roughly at the same place from the beginning until the end of the reaction to eliminate the effects of water configuration changes on the energetics. Therefore, in all cases, the explicit water molecules benefit from solvation in the reactant state and such benefit is reduced when they more strongly interact with the product (the energy of the reactants thus shifts further down than the transition states and products), thus explaining the upward shift in the reaction and activation energies. Implicit solvation is thus critical in that it removes the unphysical destabilization of the water molecules on the surface in vacuum because no other polar molecules are present near them in the reactant state for them to favorably interact with. In real solution, other water molecules are present, which the water continuum model replaces.

## Discussion

Simulations beyond DFT to compare to are available but remain few and far in between. Of note, Sautet and co-workers implemented the random phase approximation (RPA) in studying *CO diffusion,^64^ reduction,^65^ and coupling^66^ on Cu. Schimka et al. and Wei et al. showed that RPA correctly predicts the atop site to be the preferred adsorption site on Cu(111) and Cu(100), respectively, improving upon their baseline method DFT-PBE.^28,64^ With respect to coupling, Wei and Sautet calculated the RPA free energies to form coupling products, namely, OC*–*CO, OC*–*CHO, and OC*–*COH, relative to CO­(g) and (H^+^ + e^–^) on Cu(100) and showed positive free energies: ≳1 eV.^66^ When using the free energies of their implicitly solvated models (no explicit water molecules), one may calculate coupling free energies (at zero excess charge) of 1.36, 0.20, and 0.05 eV for OC*–*CO, OC*–*CHO, and OC*–*COH, respectively.^66^ Without calculating barriers, they propose that OC*–*CHO will be the dominant coupling product despite OC*–*COH having a lower coupling free energy because the former is lowest in energy relative to CO­(g), and (*CO + *CHO) is 0.48 eV lower in free energy than (*CO + *COH).^66^ We highlight that they found OC*–*CO formation (referenced to 2*COs) to be highly endoergic in RPA (1.36 eV), DFT-PBE (0.89 eV), and DFT-RPBE (0.99 eV), contrary to the results from explicitly water-solvated DFT calculations (vide supra). In ref 66, at first glance, when only looking at the free energies relative to CO­(g) and (H^+^ + e^–^), it may seem that RPA and DFT-PBE yield disparate results for the coupling reactions. Referencing the energies of the coupling products to their respective origin C_1_ species (as we have illustrated above for OC*–*CO), reveals that RPA, DFT-PBE, and DFT-RPBE in fact reasonably agree.^66^ Here, it appears that it is the lack of explicit water solvation that may explain (at least in the case of the DFT results) the larger coupling energies the authors reported compared to other DFT-based simulations.^16,19,21,22^

When using ECASPT2, we found, unlike prior DFT- and RPA-based theoretical work (vide supra), that even with mixed implicit–explicit water solvation that the nonelectrochemical coupling of two *COs to form neutral OC*–*CO is highly unfavorable (∼2 eV). Note that we were able to replicate low reaction and activation free energies for this reaction within DFT-PBE+D3BJ, therefore unlike in the RPA study discussed above,^66^ it is not the lack of explicit water (which are accounted for in DFT-based simulations in refs 16, 19, 21, and 22 ) that lead to our predicted high barrier for *CO coupling but, unambiguously, due to the use of ECASPT2 level of theory. The coupling of a *CO and *COH to form OC*–*COH^δ−^, while thermodynamically favorable (<0 eV), is also subject to a large kinetic barrier (1.2 eV). For this coupling reaction, both DFT-PBE+D3BJ and ECASPT2 qualitatively and somewhat quantitatively agree (Figure 4 and Table 1). It becomes clear from the free energetics of the formation of OC*–*COH^δ−^ that addition of electrons (here by the addition of a H atom and partial charge transfer from the surface) enables C–C coupling thermodynamically, although it remains kinetically hindered. Finally, although DFT-PBE+D3BJ predicts coupling of two *COHs to have a modest barrier, ECASPT2 predicts this reaction to have even smaller barrier (∼0.3 eV). Both levels of theory predict this reaction to be highly exothermic. However, given the large diffusion barrier for *COH (∼0.9 eV),^39^ this coupling reaction would be a rare event at low coverages (relying on random formation of nearby *COH pairs) and only would occur at high *COH coverages, a condition that precludes the need for frequent hopping of *COH and long diffusion trajectories, and thus enables formation of HOC*–*COH.

Comparing what we found for Cu(100) here and previously on Cu(111),^41^ the barrier for C–C coupling on Cu(100) is higher compared to Cu(111) using similar theoretical methods to those we employed here. On Cu(111), we made similar findings in ref 41 that the nonelectroactive coupling of *COs into OC*–*CO is less favorable than the coupling of *COH and *CHO. These species couple into bridge/bridge HOC*–*CHO, hollow­(fcc)/hollow­(hcp) HOC*–*COH, and hollow­(fcc)/hollow­(hcp) *OHC–CHO*, where the former is the most kinetically favored, with an activation free energy of only 0.3 eV. We reported an ECASPT2 free energy barrier of 0.59 eV for atop/bridge OC*–*CO in the same publication, far lower than what we calculated here (1.74 eV) for a similar species. Note that experimentally, both Cu(111) and Cu­(100) form ethylene. What differentiates the two facets is their selectivity, where Cu(100) favors ethylene over methane. Single crystal experiments by Hori et al., for example, measured CH_4_:C_2_H_4_ percent current efficiencies of 8.6:23.3 for Cu(100) and 21.8:10.2 for Cu(111) at constant voltages of −1.35 and −1.41 V vs the normal hydrogen electrode, respectively.^11^ Tentatively, we may conclude that on Cu(100), C–C coupling occurs via a different path (vide infra) and on Cu(111), C–C coupling is not the rate-determining step (given the low barriers) for ethylene formation and in fact likely *CO reduction to either *COH or *CHO is rate-limiting on Cu(111).^41^ Indeed we concluded in ref 41 that it must be the second hydrogenation step that determines selectivity of Cu(111) for methane. On Cu(100), it may be that selectivity is determined beyond the second hydrogenation step.

Finally, one may argue that because [OC*–*COH]^δ−^ forms much more easily than OC*–*CO (in fact even exothermic), the addition of an electron during coupling of two *COs may ease the formation of an equivalent deprotonated species [OC*–*CO]^(1+δ)–^, i.e., electroactive coupling. This was, after all, what Goodpaster et al.,^18^ Garza et al.,^20^ Montoya et al.,^16^ and Kristoffersen and Chan^21^ showed all within DFT. In a companion paper, we show that while this also happens to be the case for ECASPT2, the reaction to form *COH from *CO is just overwhelmingly faster than the electroactive *CO coupling such that *COH formation will easily deplete *CO at relevant reducing potentials, making the formation of [OC*–*CO]^(1+δ)–^ noncompetitive.^67^

## Conclusions

We showed that within the ECASPT2 formalism the coupling of *CO with a *CO or a *COH is kinetically hindered, contrary to prior DFT-based simulations. We also found the coupling of two *COHs at adjacent hollow sites to have a surmountable barrier at room temperature and to be highly exoergic. However, because of the previously predicted low surface mobility of *COH,^39^ formation of HOC*–*COH will be limited to high *COH coverages. We therefore conclude that *CO and the favored one-electron reduction product *COH are unlikely to participate in the nonelectroactive C–C bond formation on pristine Cu(100). Given current predictions and our previous bodies of work, it is clear that the field should shift focus from *CO, *COH, and *CHO to more hydrogenated species, such as *CH_*x*_OH and *CH_*x*_, in identifying the key species that enable C–C bond formation at moderately negative potentials, which then leads to C_2+_ product formation during ECO/CO_2_R on Cu(100).

## Supplementary Material



## Notes

ECASSCF active space initialization procedure; atomic structures and relative energies of different adsorption configurations for OC*–*CO, OC*–*COH, and HOC*–*COH; illustration of which atoms were included in the vibrational free energy calculation; active space natural orbitals and occupation; AS size convergence test for the ECASPT2 reaction energy curve for atop/atop OC*–*CO formation in a vacuum; structures along the path to form bridge/bridge – parallel OC*–*CO optimized in a vacuum; structural reference gas-phase molecules; reaction and activation internal energies without ZPE (PDF)
