# Supplementary material for: Insights into Nonelectroactive C–C Bond Formation on Cu(100) during Electrochemical CO2 Reduction from Multiconfigurational Wavefunction Theory
Source: J Phys Chem C Nanomater Interfaces. 2026 Feb 27;130(10):3767–79. doi: 10.1021/acs.jpcc.5c07792 (PMC12990116; doi:10.1021/acs.jpcc.5c07792)
Supplement: Supplementary file 1 [file jp5c07792_si_001.pdf]

Supplementary Information

Insights into Non-Electroactive C-C Bond Formation on Cu(100) During Electrochemical CO_2 Reduction from Multiconfigurational Wavefunction Theory

John Mark P. Martirez[1,*] and Emily A. Carter[1,2,*]
[1]Applied Materials and Sustainability Sciences, Princeton Plasma Physics Laboratory, Princeton, New Jersey, 08543-0451, United States
[2]Department of Mechanical and Aerospace Engineering and the Andlinger Center for Energy and the Environment, Princeton University, Princeton, New Jersey 08544-5263, United States
*Corresponding authors: martirez@pppl.gov, eac@princeton.edu

Supplementary Methods

CASSCF active space initialization. To generate the initial guess orbitals of the AS, we applied techniques described in ref. 1. Specifically, we performed CASSCF with the (vacuum-optimized) adsorbates shifted 10 Å away from the surface side of the cluster where we optimized first the orbitals for the separated product adsorbate structure and the metal cluster. We then used the optimized natural orbitals of the desorbed products (**Figs. S6-S9,** left panels) to initialize the orbitals of the molecules (still 10 Å away from the surface side of the cluster) of the structure that comes just before it along the reaction coordinate. At this stage, since the molecules are far away from the cluster, we optimized the orbitals without V_{emb}. We continue with this procedure along the reaction coordinate ("creeping") until we generate the optimized natural orbitals of the desorbed reactant structures (**Figs. S10-S12,** left panels). Note that the optimized orbitals of the Cu cluster are unchanged throughout the procedure because the cluster does not change and is far away from the molecules. Finally, we use the natural orbitals of the desorbed molecules and metal cluster (one set for each image along the reaction pathway) to initialize the orbitals of the combined adsorbate and cluster structures ("orbital merging") and optimize them now in the presence of V_{emb} (**Figs. S6-S12,** center and right panels). The captions of **Figs. S6-S12** discusses the rationale for the chosen AS sizes and the nature of the AS orbitals. We did not repeat the same procedure for the solvent-optimized structures. Instead, we used the optimized ECASSCF natural orbitals of the vacuum-optimized structures (with the molecules adsorbed on the cluster) to initialize the orbitals of the solvent-optimized structures within ECASSCF.

Supplementary Figures

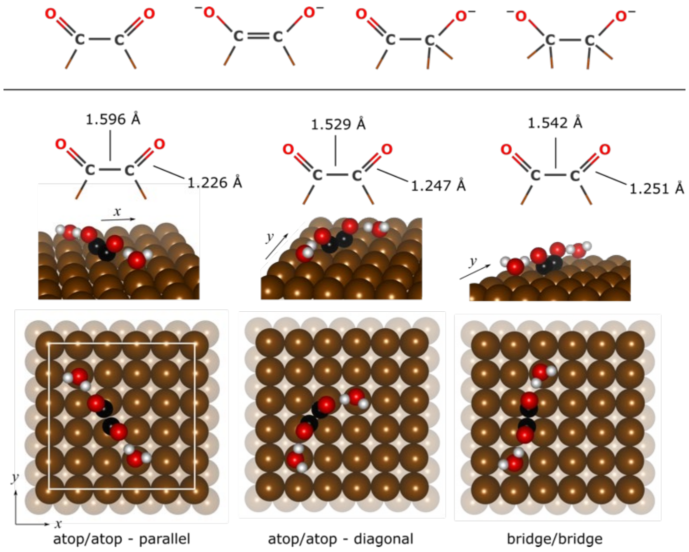

Figure S1. First row, Lewis structures of possible OC*–*CO bonding motifs and charge states. Second row, we ascribed the most representative Lewis structures of the DFT-PBE+D3BJ vacuum-optimized structures based on their C-C and C-O bond lengths (annotated). Third and fourth rows, side and top views of the optimized OC*–*CO + $2H_2O$ structures with the two explicit solvating water molecules hydrogen bonded to the carbonyl O atoms. Cu – dark brown spheres, C – black, O – red, and H – white. Vectors: *x* = $[011]$, *y* = $[0\bar{1}1]$, *z* = $[100]$. For the top views, the white box bounds the perimeter of the (5×5) supercell (shown only for one structure).

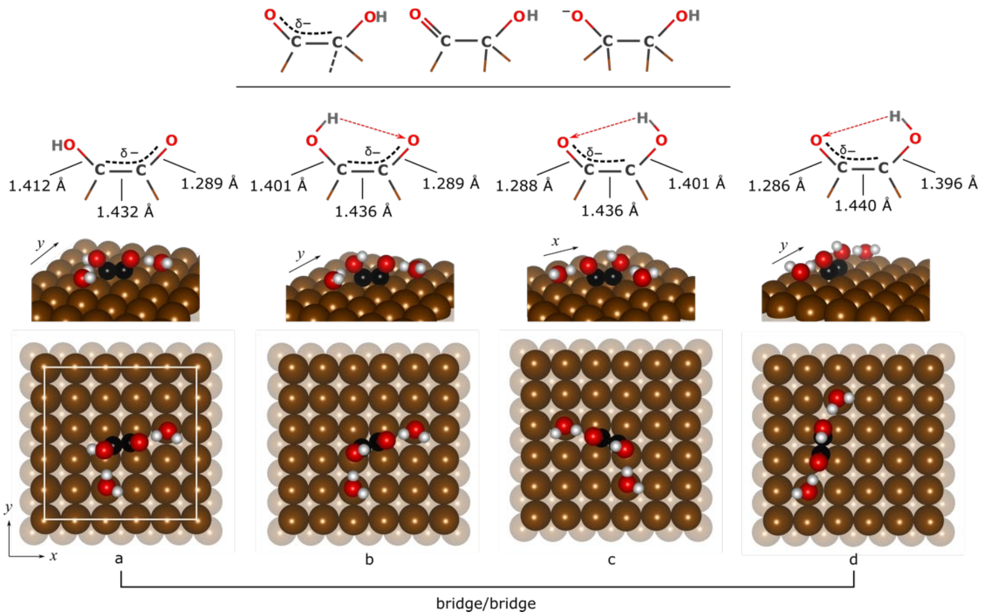

Figure S2. First row, Lewis structures of possible OC*–*COH bonding motifs and charge states. Second row, we ascribed the most representative Lewis structures of the DFT-PBE+D3BJ vacuum-optimized structures based on their C-C and C-O bond lengths (annotated). Third and fourth rows, side and top views of the optimized OC*–*COH + $2H_2O$ structures with the two explicit solvating water molecules hydrogen bonded to the carbonyl and hydroxyl O atoms. Cu – dark brown spheres, C – black, O – red, and H – white. Vectors: $x = [011]$, $y = [0\bar{1}1]$, $z = [100]$. For the top views, the white box bounds the perimeter of the (5×5) supercell (shown only for one structure). Fig. 2 in the main text elaborates the meaning of delocalized negative charge picture. The C-C and C-O bond lengths supporting the partial anionic character picture. Red dash arrows show internal hydrogen bonding pointing from donor to acceptor.

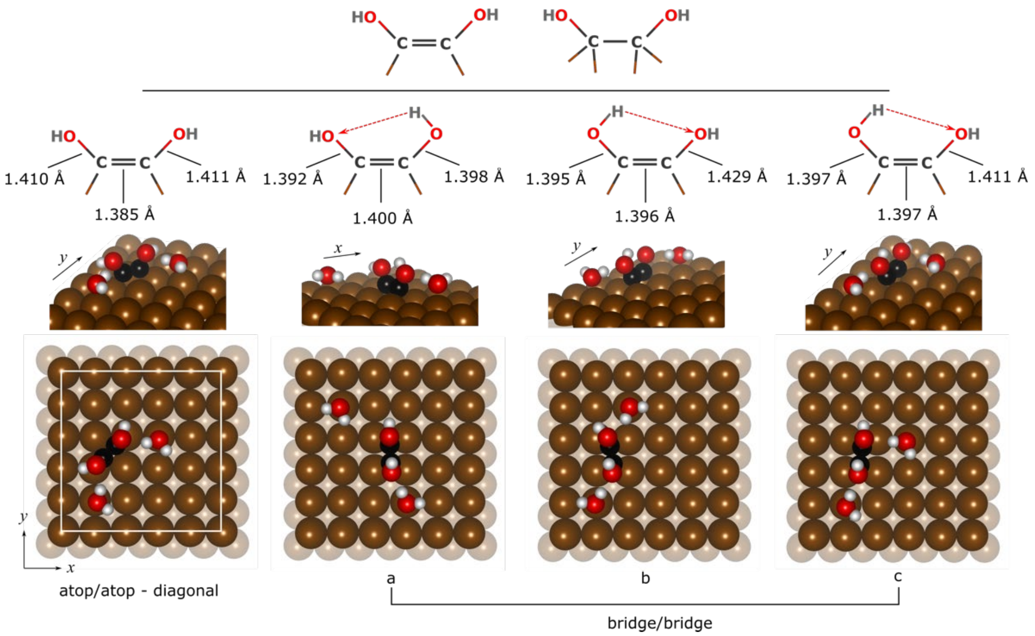

Figure S3. First row, Lewis structures of possible HOC*–*COH bonding motifs and charge states. Second row, we ascribed the most representative Lewis structures of the DFT-PBE+D3BJ vacuum-optimized structures based on their C-C and C-O bond lengths (annotated). Third and fourth rows, side and top views of the optimized HOC*–*COH + $2H_2O$ structures with the two explicit solvating water molecules hydrogen bonded to the hydroxyl O atoms. Cu – dark brown spheres, C – black, O – red, and H – white. Vectors: *x* = $[011]$, *y* = $[0\bar{1}1]$, *z* = $[100]$. For the top views, the white box bounds the perimeter of the (5×5) supercell (shown only for one structure). Red dash arrows show internal hydrogen bonding pointing from donor to acceptor.

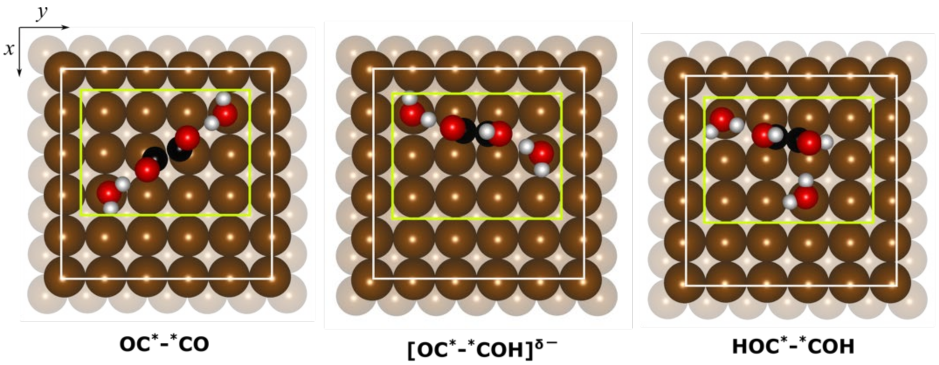

Figure S4. Top views of the ECASPT2-favored structures illustrating which atoms were included in the construction of the Hessian in the vibrational free energy calculation for the adsorbates. The neon green rectangles bound the 12 Cu atoms along with the two water molecules and adsorbates (as labelled). The bounding box contains all the Cu atoms that directly participate along the coupling reaction pathway. Cu – dark brown spheres, C – black, O – red, and H – white. Vectors: *x* = $[011]$, *y* = $[0\bar{1}1]$, *z* = $[100]$. The white boxes bound the perimeter of the (5×5) supercells.

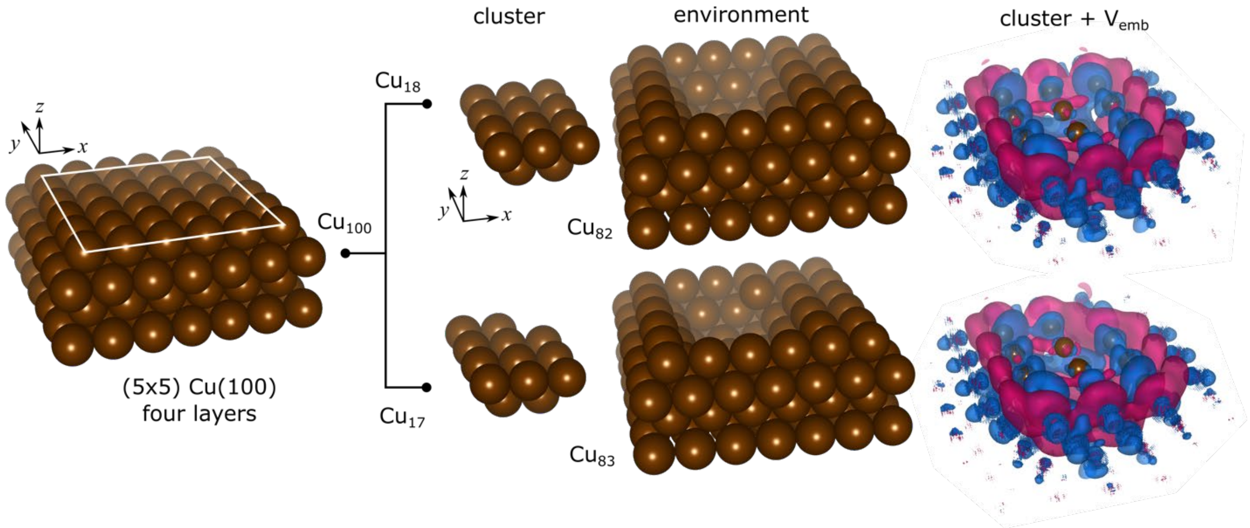

Figure S5. Partitioning scheme and embedding potentials. Full four-layer (5×5) supercell Cu(100) slab (first column) and two types of clusters (second column) and environment (third column) structures used herein. Fourth column, optimized embedding potentials for the two partitioning schemes. Iso-surface shows attractive potentials (−1.5 V, pink) at the partitioning boundaries and repulsive potentials (+1.5 V, light blue) at and near the core of the boundary atoms. The embedding potential simulates the missing metallic bonding interactions between the cluster and its environment. Vectors: x = $[011]$, y = $[0\bar{1}1]$, z = $[100]$. The white box in the full structure bounds the perimeter of the (5×5) supercell.

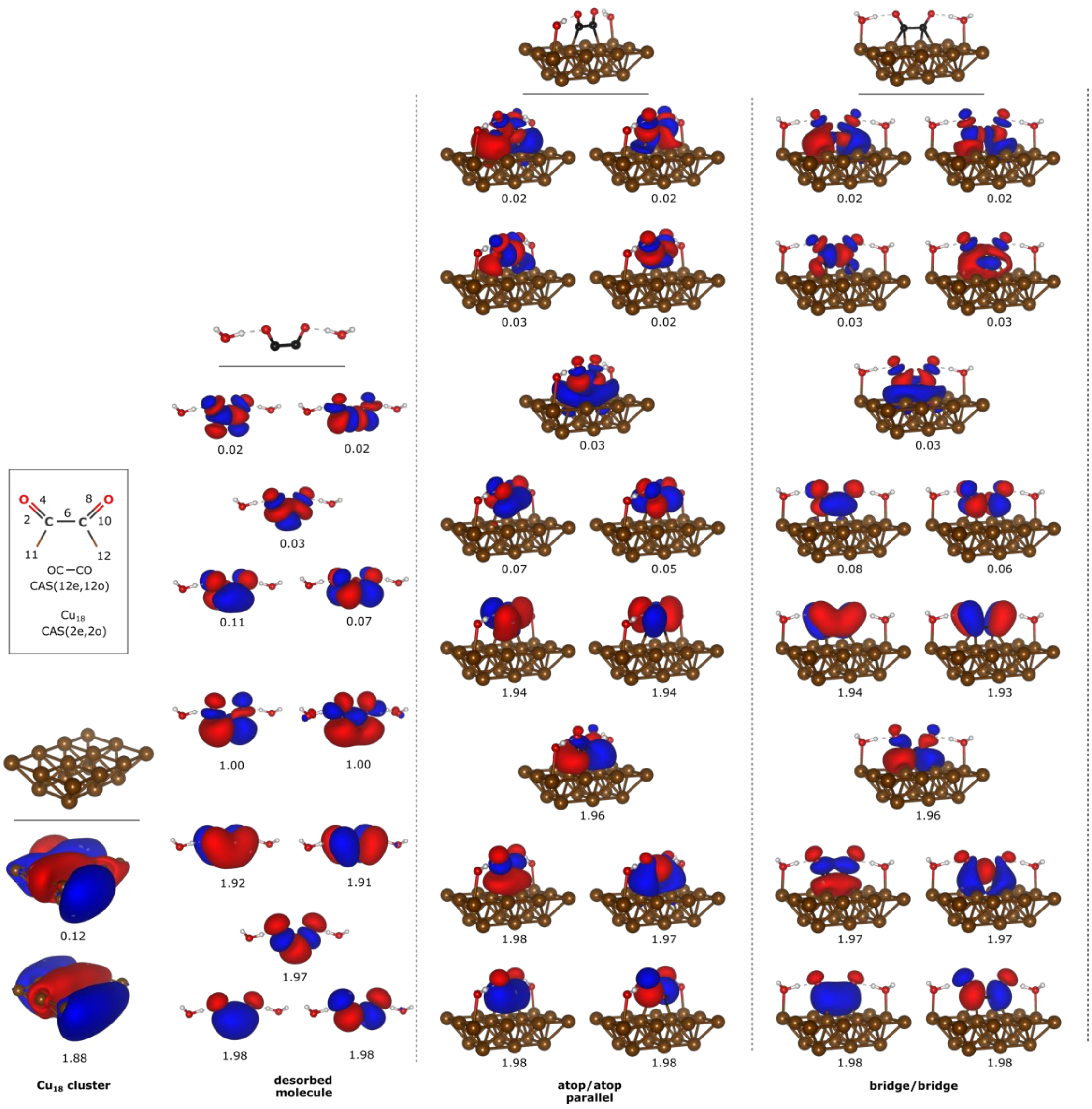

Figure S6. Optimized ECASSCF AS(14e,14o) natural orbitals and occupation numbers for OC*–*CO with $2H_2O$. On top of each column of orbitals are the corresponding structures (without orbitals). Left panel, natural orbitals (with occupation numbers) of an adsorbate-free embedded Cu_{18} cluster and isolated OC*–*CO that were used to initialize the AS for adsorbed OC*–*CO on the same cluster. Top left inset shows the electron and orbital counting scheme we employed to determine the AS size. We count the number of pertinent bonds (and corresponding electrons), namely, C-O (8e,8o) and C-C (2e,2o), the "dangling" orbitals in C atoms (2e,2o), and Cu orbitals needed to facilitate bonding between Cu and C atoms (2e,2o), yielding a total of (14e,14o). The isolated OC*–*CO AS includes the two sets of C-O σ, π, σ^*, and π^*, C-C σ and σ^*, and two singly occupied non-bonding (dangling) C 2*s*- and 2*p*-derived orbitals. Center panel, optimized AS for the atop/atop – parallel configuration (**Fig. S1**) that shows the same bonding interactions in the isolated molecule but now features the bonding and antibonding orbitals associated with C-Cu bonding. Right panel, optimized AS for the bridge/bridge configuration showing similar orbitals as in the atop/atop – parallel configuration (**Fig. S1**).

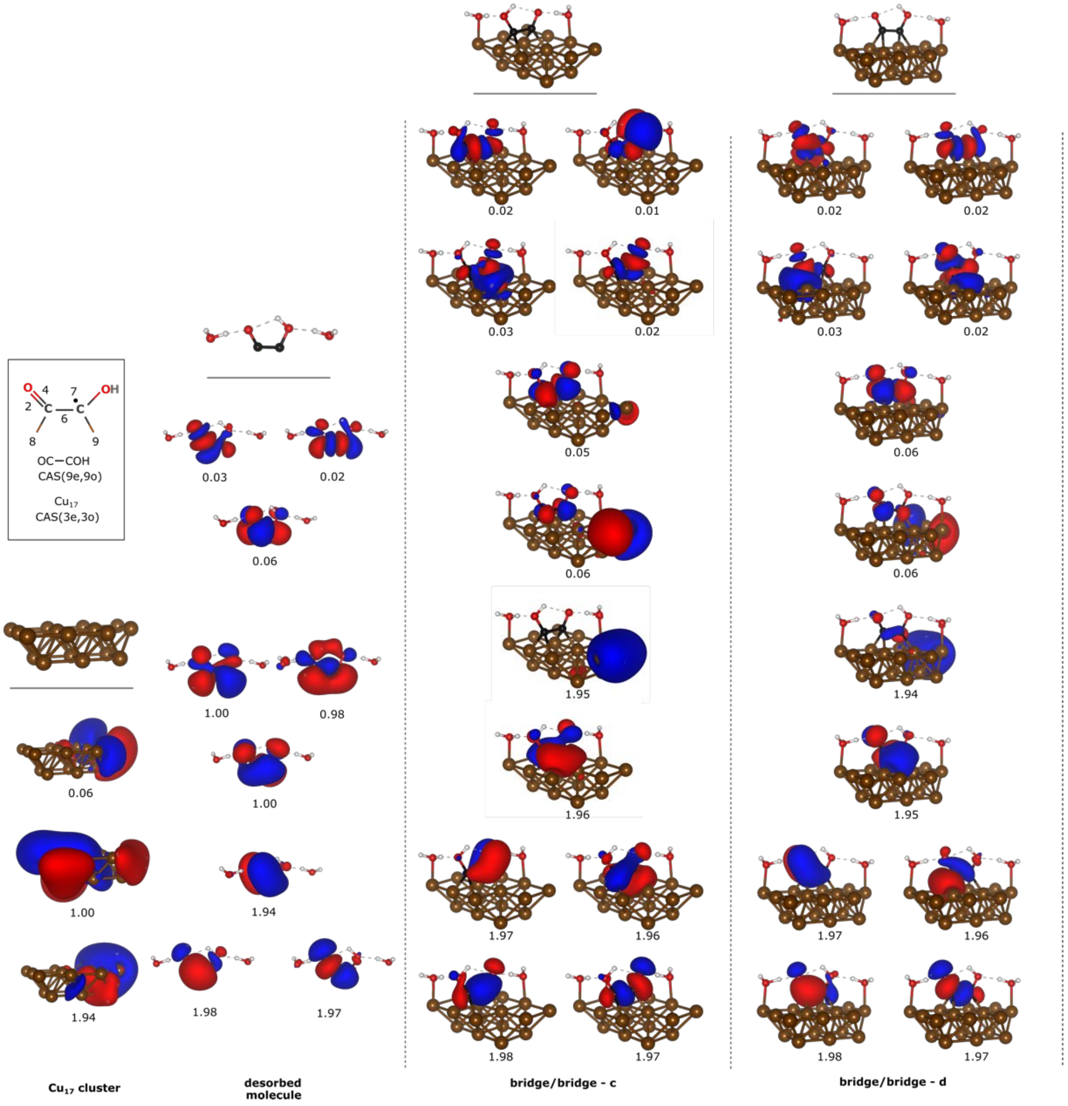

Figure S7. Optimized ECASSCF AS(12e,12o) natural orbitals and occupation numbers for OC*–*COH with $2H_2O$. On top of each column of orbitals are the corresponding structures (without orbitals). Left panel, natural orbitals (with occupation numbers) of an adsorbate-free embedded Cu_{17} cluster and isolated OC*–*COH that were used to initialize the AS for adsorbed OC*–*COH on the same cluster. Top left inset shows the electron and orbital counting scheme we employed to determine the AS size. We count the number of pertinent bonds (and corresponding electrons), namely, carbonyl C-O (4e,4o) and C-C (2e,2o), the "dangling" orbitals in C atoms (3e,3o), and Cu orbitals needed to facilitate bonding between Cu and C atoms (3e,3o), yielding a total of (12e,12o). The isolated OC*–*COH AS includes the carbonyl C-O σ, π, σ^*, and π^*, C-C σ and σ^*, and three singly occupied non-bonding (dangling) C 2*s*- and 2*p*-derived orbitals. Center panel, optimized AS for the bridge/bridge – c configuration (**Fig. S2**) that shows the same bonding interactions in the isolated molecule but now features the bonding and antibonding orbitals associated with C-Cu bonding and an occupied-unoccupied orbital pair from the Cu cluster. Right panel, optimized AS for the bridge/bridge – d configuration (**Fig. S2**) showing similar orbitals as in the bridge/bridge – c configuration.

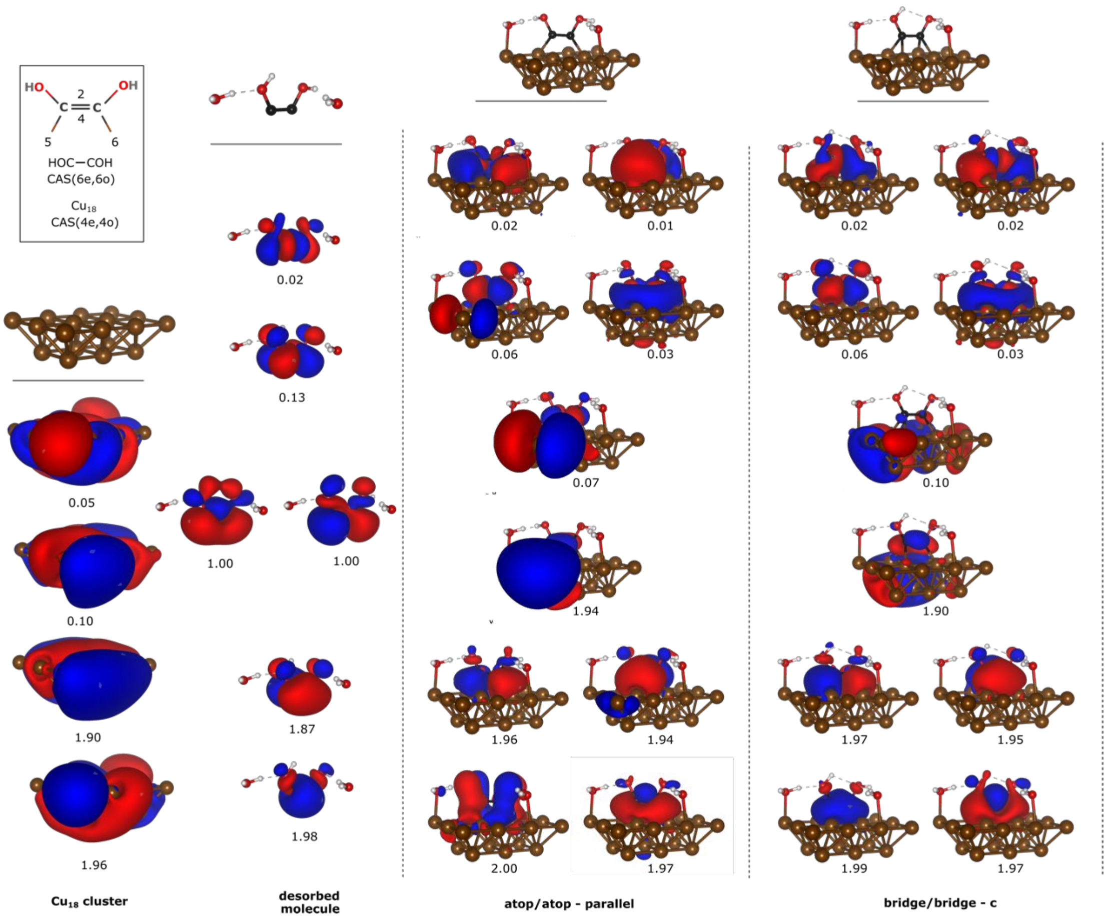

Figure S8. Optimized ECASSCF AS(10e,10o) natural orbitals and occupation numbers for HOC*–*COH with 2H_2O. On top of each column of orbitals are the corresponding structures (without orbitals). Left panel, natural orbitals (with occupation numbers) of an adsorbate-free embedded Cu_{18} cluster and isolated HOC*–*COH that were used to initialize the AS for adsorbed HOC*–*COH on the same cluster. Top left inset shows the electron and orbital counting scheme we employed to determine the AS size. We count the number of pertinent bonds (and corresponding electrons), namely, C-C (4e,4o), the "dangling" orbitals in C atoms (2e,2o), and Cu orbitals needed to facilitate bonding between Cu and C atoms (2e,2o) with an additional (2e,2o) to allow for two possible Cu-C bonds per C at the bridge site if needed, thus yielding a total of (10e,10o). The isolated HOC*–*COH AS includes the C-C σ, π, σ^*, and π^*, and two singly occupied non-bonding (dangling) C 2*s*- and 2*p*-derived orbitals. Center panel, optimized AS for the atop/atop – parallel configuration (**Fig. S3**) that shows the same bonding interactions in the isolated molecule but now features the bonding and antibonding orbitals associated with C-Cu bonding and an occupied-unoccupied orbital pair from the Cu cluster. Right panel, optimized AS for the bridge/bridge – c configuration (**Fig. S3**) showing similar orbitals as in the atop/atop – parallel configuration. We use an AS of (12e,12o) for the coupling reaction to accommodate all six Cu-C σ, σ^* pairs for the two monomeric *COHs at the reactant (**Figs. S9** and **S12**).

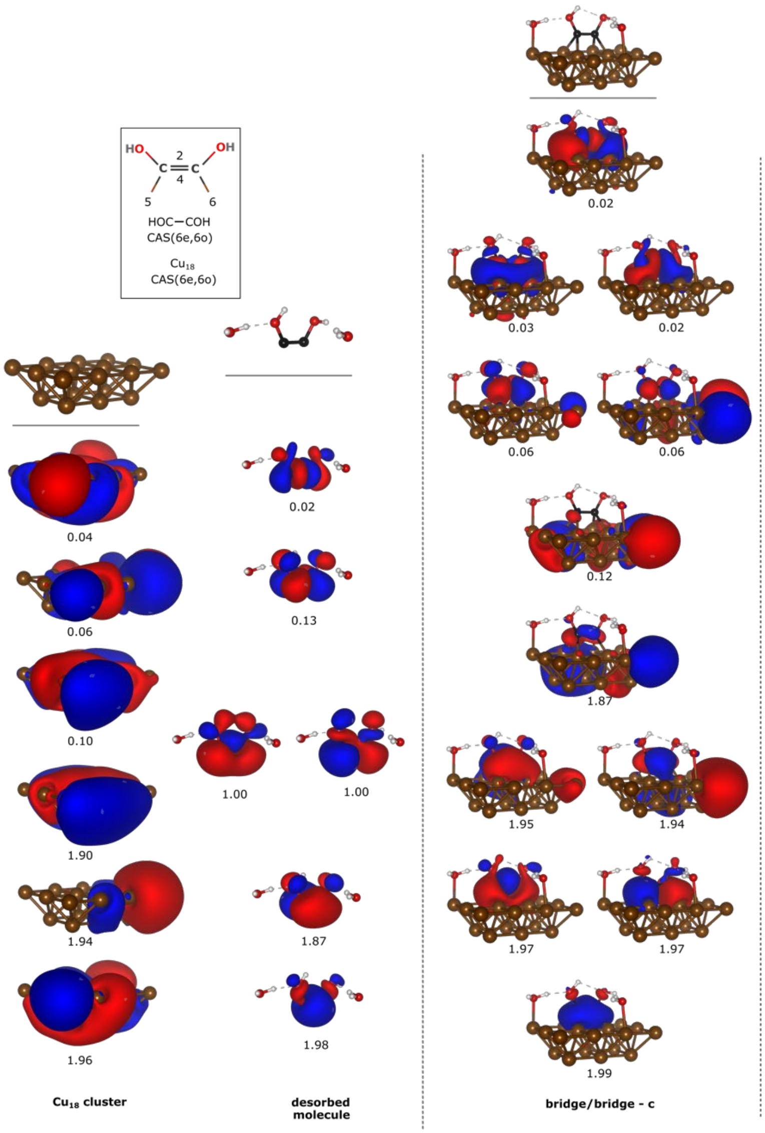

Figure S9. Optimized ECASSCF AS(12e,12o) natural orbitals and occupation numbers for HOC*–*COH with $2H_2O$. On top of each column of orbitals are the corresponding structures (without orbitals). Left panel, natural orbitals (with occupation numbers) of an adsorbate-free embedded Cu_{18} cluster and isolated HOC*–*COH that were used to initialize the AS for adsorbed HOC*–*COH on the same cluster. Top left inset shows the electron and orbital counting scheme we employed to determine the AS size. We count the number of pertinent bonds (and corresponding electrons), namely, C-C (4e,4o), the "dangling" orbitals in C atoms (2e,2o), and Cu orbitals needed to facilitate bonding between Cu and C atoms (2e,2o) with additional (4e,4o) to accommodate all six Cu-C σ, σ^* pairs for the two monomeric *COHs at the reactant (**Fig. S12**), thus yielding a total of (12e,12o). As in **Fig. S8**, the isolated HOC*–*COH AS includes the C-C σ, π, σ^*, and π^*, and two singly occupied non-bonding (dangling) C 2*s*- and 2*p*-derived orbitals. Right panel shows the optimized AS for the favored coupling product bridge/bridge – c configuration (**Fig. S3**). AS(12e,12o) exhibits a similar set of orbitals as in AS(10e,10o) in **Fig. S8**, except for an additional pair of occupied and empty Cu 4*s*-derived states mixed in with some C-C π– and π^*-like states.

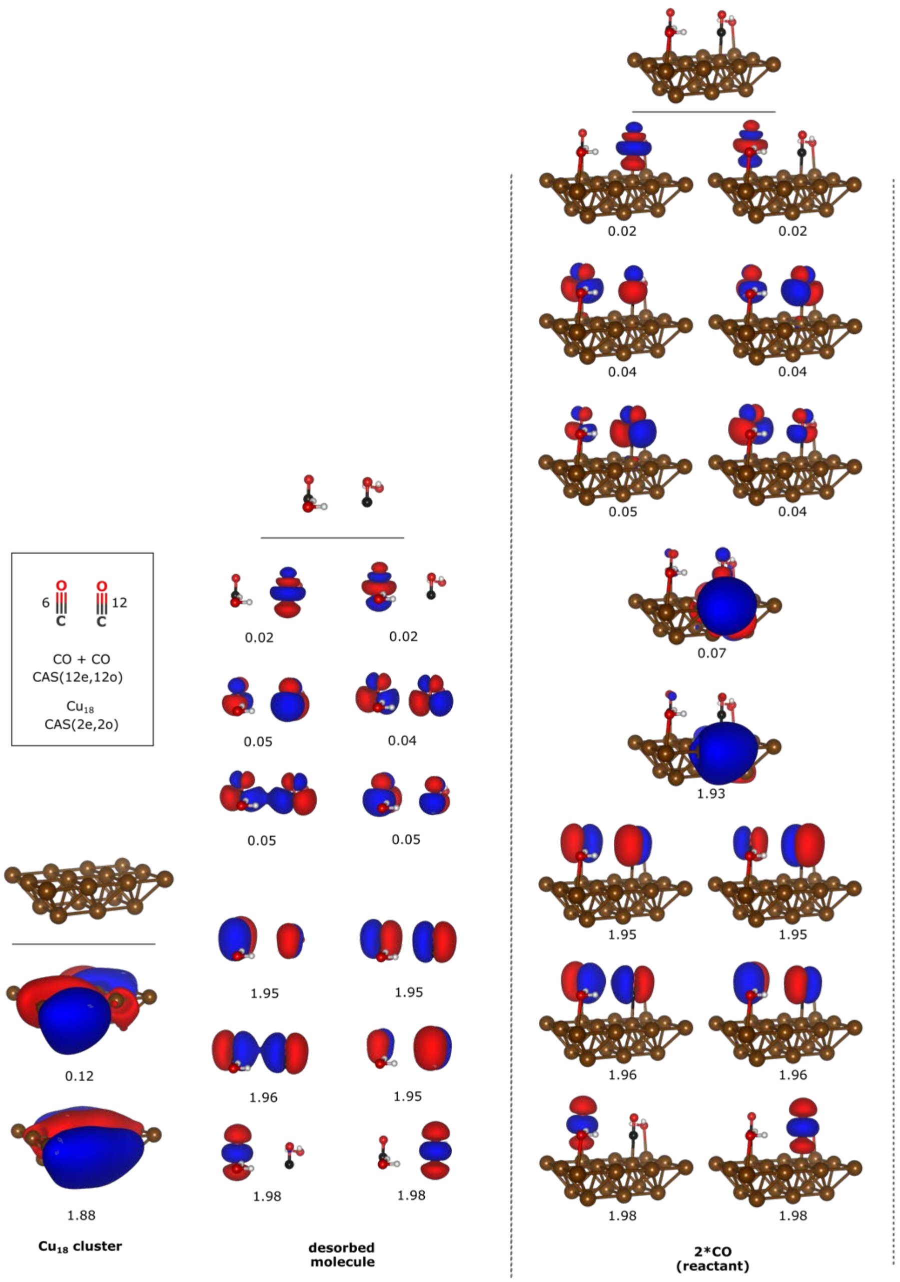

Figure S10. Optimized ECASSCF AS(14e,14o) natural orbitals and occupation numbers for the reactant (atop *CO + atop *CO with $2H_2O$) leading to atop/atop – parallel OC*–*CO. On top of each column of orbitals are the corresponding structures (without orbitals). Left panel, natural orbitals (with occupation numbers) of an adsorbate-free embedded Cu_{18} cluster and isolated *CO + *CO that were used to initialize the AS for adsorbed *CO + *CO on the same cluster. Top left inset shows the electron and orbital counting scheme we employed to determine the AS size. The isolated *CO + *CO orbitals were initialized using the "creeping" method (Supplementary Methods) along the reaction path (using the structures along the reaction of the adsorbed species) starting from the isolated OC*–*CO (**Fig. S6**, left panel). The isolated *CO + *CO AS includes the C-O σ, π, σ^*, and π^* orbitals of the two *CO molecules. Right panel, optimized AS for the atop + atop configuration that shows the same bonding interactions in the isolated molecules but now features an occupied-unoccupied orbital pair from the Cu.

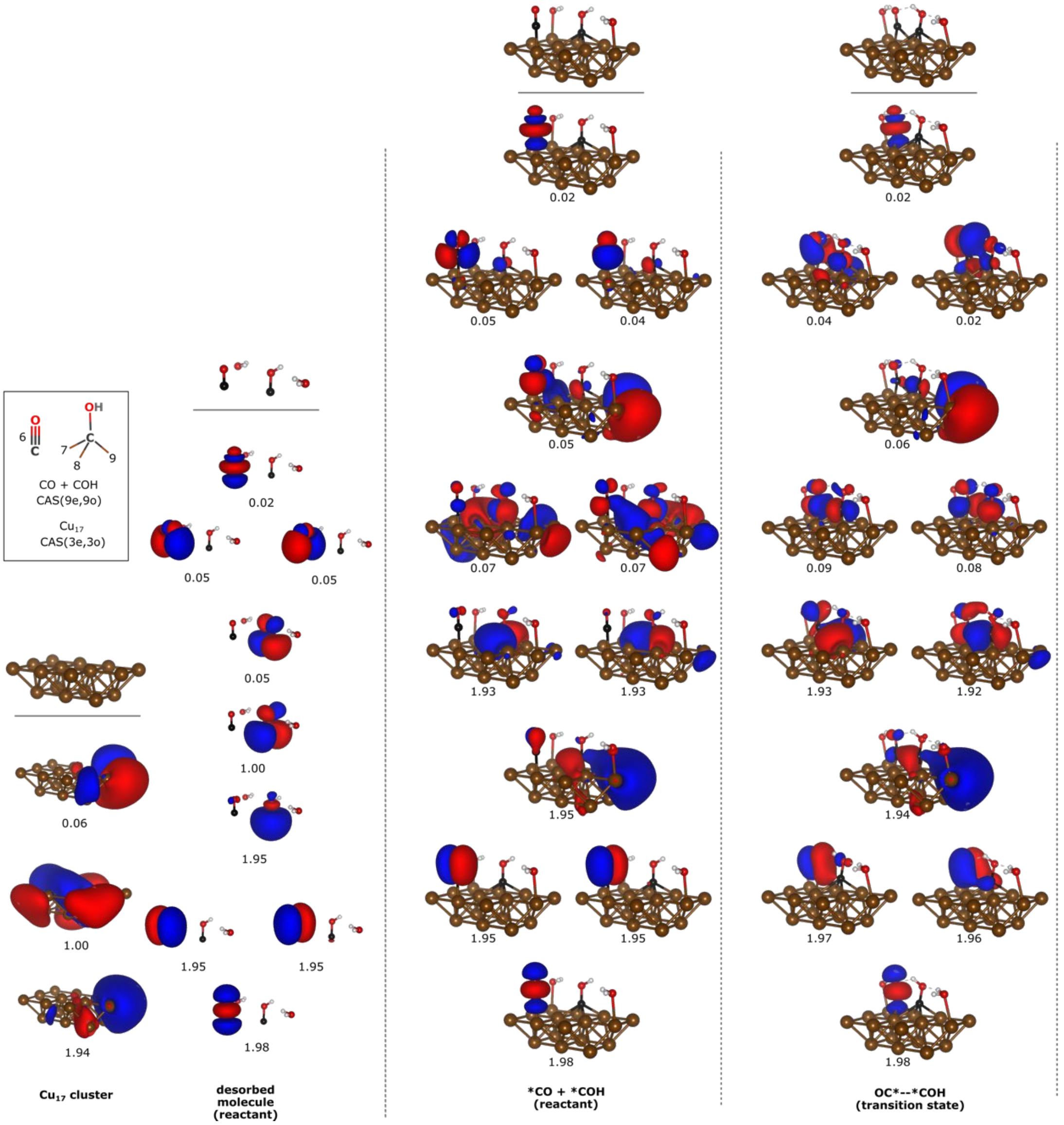

Figure S11. Optimized ECASSCF AS(12e,12o) natural orbitals and occupation numbers for the reactant (atop *CO + hollow *COH with $2H_2O$) and transition state leading to bridge/bridge OC*–*COH. On top of each column of orbitals are the corresponding structures (without orbitals). Left panel, natural orbitals (with occupation numbers) of an adsorbate-free embedded Cu_{18} cluster and isolated *CO + *COH that were used to initialize the AS for adsorbed *CO + *COH on the same cluster. Top left inset shows the electron and orbital counting scheme we employed to determine the AS size. The isolated *CO + *COH orbitals were initialized using the "creeping" method (Supplementary Methods) along the reaction path (using the structures along the reaction of the adsorbed species) starting from the isolated OC*–*COH (**Fig. S7**, left panel). The isolated *CO + *COH AS includes the C-O σ, π, σ^*, and π^* orbitals of *CO and three non-bonding C 2*s*- and 2*p*-derived orbitals of *COH. Center panel, optimized AS for the atop + hollow configuration showing similar *CO orbitals but now features bonding and antibonding orbitals associated with C-Cu bonding originating from *COH and an occupied-unoccupied orbital pair from the Cu. Right panel, optimized AS for the transition state exhibiting some mixing of the *CO- and *COH-derived orbitals although, as expected, clear C-C σ and σ^* bonding orbitals (**Fig. S7**, right panel) have yet to emerge.

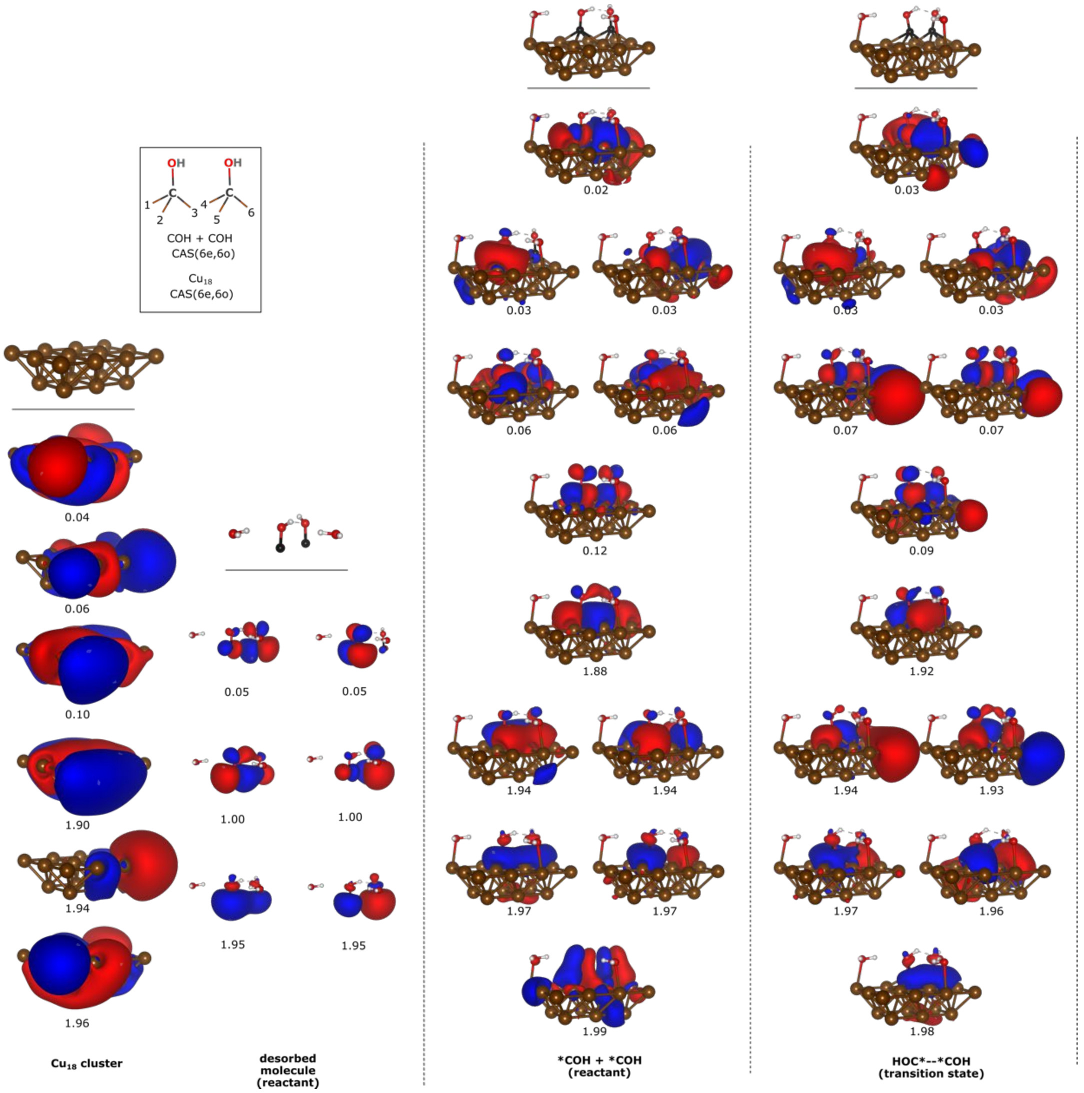

Figure S12. Optimized ECASSCF AS(12e,12o) natural orbitals and occupation numbers for the reactant (hollow *COH + hollow *COH with $2H_2O$) leading to bridge/bridge HOC*–*COH. On top of each column of orbitals are the corresponding structures (without orbitals). Left panel, natural orbitals (with occupation numbers) of an adsorbate-free embedded Cu_{18} cluster and isolated *COH + *COH that were used to initialize the AS for adsorbed *COH + *COH on the same cluster. Top left inset shows the electron and orbital counting scheme we employed to determine the AS size. The isolated *COH + *COH orbitals were initialized using the “creeping” method (Supplementary Methods) along the reaction path (using the structures along the reaction of the adsorbed species) starting from the isolated HOC*–*COH (**Fig. S9**, left panel). The isolated *COH + *COH AS includes six non-bonding C 2*s*- and 2*p*-derived orbitals of the two *COH molecules, matched with AS(6e,6o) in the Cu_{18} cluster to form the six Cu–C bonds. Center panel, optimized AS for the hollow + hollow configuration that shows all six pairs of Cu–C $\sigma,\sigma*$ orbitals. Right panel, optimized AS for the transition state already exhibiting occupied C–C σ- and π-bonding-like and empty $\sigma*$- and $\pi*$-bonding-like orbitals.

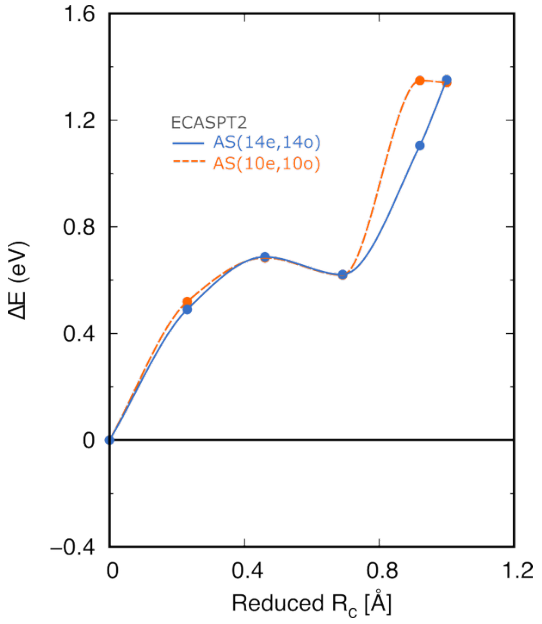

Figure S13. AS size convergence of the ECASPT2 potential energy curve for the reaction $2^{*}CO$(atop) → $OC^{*}–^{*}CO$ (atop/atop) in vacuum. Potential energies along the coupling path (**Fig. 3A**) from AS of (10e,10o) and (14e,14o) are shown. The transition state predicted by DFT-PBE+D3BJ is notably stabilized by 0.25 eV relative to the product going from (10e,10o) to (14e,14o). All other energy differences between the two ASs are 0.03 eV or less, specifically 0.01 eV at the product.

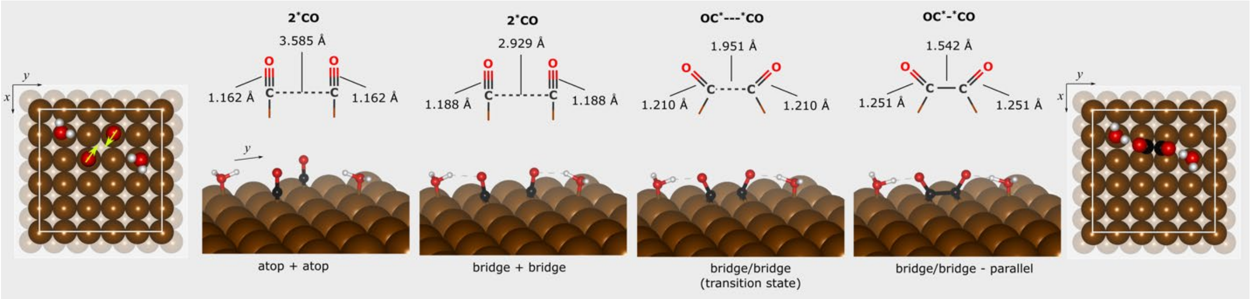

Figure S14. DFT-PBE+D3BJ vacuum-optimized structures along the minimum-energy coupling paths for the formation of bridge/bridge – parallel OC*-*CO starting from two atop *COs. The two (top and side views) leftmost and two (side and top views) rightmost panels are the reactant and product. The structures in between the reactants and products are either the transition state (as marked) or a local minimum. The structures are labelled according to the adsorption site of the *COs. If the species are uncoupled or coupled, the label shows "+" or "/", respectively. The structures shown here correspond to the data points marked with red circles in the reaction energy curve in **Fig. 4A** in the main text. For the top views, the white boxes show the boundary of the periodic (5×5) supercells and on the reactant, the neon green arrows chart schematically the trajectory of the molecules as they form the product. The C-C and C-O distances are annotated in their corresponding Lewis structures. Cu – dark brown spheres, C – black, O – red, and H – white. Vectors: *x* = $[011]$, *y* = $[0\bar{1}1]$, *z* = $[100]$.

Supplementary Tables

Table S1. Calculated relative energies (eV) of different OC*–*CO (+ $2H_2O$) configurations from DFT-PBE+D3BJ and ECASPT2 in vacuum.[a]

Method	atop/atop – parallel	atop/atop – diagonal	bridge/bridge
DFT-PBE+D3BJ	0.43	0.23	0.00
ECASPT2	0.00	0.46	0.44

[a]structures shown in **Fig. S1**

Table S2. Calculated relative energies (eV) of different OC*–*COH (+ $2H_2O$) configurations from DFT-PBE+D3BJ and ECASPT2 in vacuum.[a]

Method	bridge/bridge			
	a	b	c	d
DFT-PBE+D3BJ	0.27	0.12	0.11	0.00
ECASPT2	-	-	0.00	0.01

[a]structures shown in **Fig. S2**

Table S3. Calculated relative energies (eV) of different HOC*–*COH (+ $2H_2O$) configurations from DFT-PBE+D3BJ and ECASPT2 in vacuum.[a]

Method	atop/atop – diagonal	bridge/bridge		
		a	b	c
DFT-PBE+D3BJ	0.31	0.08	0.05	0.00
ECASPT2	0.19	-	-	0.00

[a]structures shown in **Fig. S3**

Table S4. Equilibrium (0 K) C-C and C-O bond lengths in select C_2 gaseous (oxygenated) hydrocarbons calculated via DFT-PBE+D3BJ, as structural references for C_2 adsorbates.

Molecule[a]	Lewis structure[b]	C-C bond length [Å]	C-O bond length [Å]
trans-glyoxal	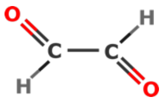	1.527	1.217 (C=O)
trans-glycolaldehyde	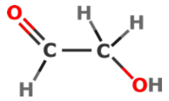	1.515	1.217 (C=O)<br>1.425 (C-OH)
trans-1,2-ethendiol	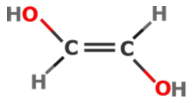	1.338	1.386 (C-OH)
anti-ethylene glycol	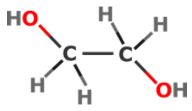	1.520	1.432 (C-OH)
cis-glyoxal	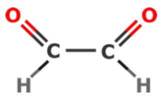	1.546	1.212 (C=O)
cis-glycolaldehyde	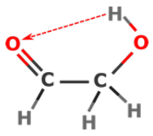	1.503	1.223 (C=O)<br>1.403 (C-OH)
cis-1,2-ethendiol	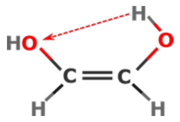	1.340	1.393 (C-OH)<br>1.370 (C-OH)
gauche-ethylene glycol	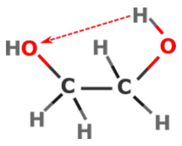	1.516	1.439 (C-OH)<br>1.427 (C-OH)
Ethene	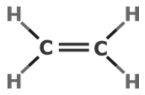	1.333	-
Ethane	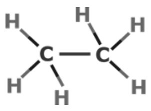	1.529	-

[a]cis and gauche are lower in energy than trans and anti, respectively, except for glyoxal, because cis and gauche enable intramolecular hydrogen bonding when the terminal moieties can do so. The C_2 adsorbates that bond through the C atoms adopt structures similar to the cis and gauche conformers. [b]Intramolecular hydrogen bonding for molecules with OH, if present, is illustrated using red dashed arrows.

Table S5. Reaction energetics (w/o ZPE) in vacuum and continuum water (ε = 78.4).

Reaction[a]	ΔE_{rxn} [eV]		$E^{\ddagger}$ [eV]	
	DFT-PBE+D3BJ	ECASPT2	DFT-PBE+D3BJ	ECASPT2
	**vacuum**			
$2^*CO \rightarrow OC^*–^*CO$ (atop/atop)	0.36	1.35	0.41	1.35[b]
$^*CO + ^*COH + Cu_4 \rightarrow [OC^*–^*COH]^{\delta-} + Cu_4^{\delta+}$	-0.89	-0.68	0.46	0.81
$2^*COH \rightarrow HOC^*–^*COH$	-1.36	-1.61	0.56	0.24
	**continuum water (ε = 78.4)**			
$2^*CO \rightarrow OC^*–^*CO$ (atop/atop)	0.60	1.66	[c]	1.66[b]
$^*CO + ^*COH + Cu_4 \rightarrow [OC^*–^*COH]^{\delta-} + Cu_4^{\delta+}$	-0.53	-0.30	0.83	1.16
$2^*COH \rightarrow HOC^*–^*COH$	-1.20	-1.38	0.60	0.29

[a]we explicitly include $Cu_4^{\delta+}$ in $[OC^*–^*COH]^{\delta-}$ for charge balance; [b]the same as ΔE_{rxn}; [c]CI-NEB not performed

Supplementary References

(1) Wen, X.; Boyn, J.-N.; Martirez, J. M. P.; Zhao, Q.; Carter, E. A. Strategies to Obtain Reliable Energy Landscapes from Embedded Multireference Correlated Wavefunction Methods for Surface Reactions. *Journal of Chemical Theory and Computation* **2024**, *20* (14), 6037-6048.
